# Adaptation of a quality improvement approach to implement eScreening in VHA healthcare settings: innovative use of the Lean Six Sigma Rapid Process Improvement Workshop

**DOI:** 10.1186/s43058-021-00132-x

**Published:** 2021-04-07

**Authors:** James O. E. Pittman, Borsika Rabin, Erin Almklov, Niloofar Afari, Elizabeth Floto, Eusebio Rodriguez, Laurie Lindamer

**Affiliations:** 1 VA Center of Excellence for Stress and Mental Health, 3350 La Jolla Village Dr., San Diego, CA USA; 2 Department of Psychiatry, University of California San Diego, 9500 Gilman Dr., La Jolla, CA USA; 3 VA San Diego Healthcare System, 3350 La Jolla Village Dr., San Diego, CA USA; 4 UC San Diego Dissemination and Implementation Science Center, University of California San Diego, 9500 Gilman Dr., La Jolla, CA USA; 5 Department of Family Medicine and Public Health, University of California San Diego, 9500 Gilman Dr., La Jolla, CA USA; 6 VA Roseburg Health Care System, 913 NW Garden Valley Blvd, Roseburg, OR USA

**Keywords:** Electronic screening, Implementation, RPIW, Quality improvement, eScreening

## Abstract

**Background:**

The Veterans Health Administration (VHA) developed a comprehensive mobile screening technology (eScreening) that provides customized and automated self-report health screening via mobile tablet for veterans seen in VHA settings. There is agreement about the value of health technology, but limited knowledge of how best to broadly implement and scale up health technologies. Quality improvement (QI) methods may offer solutions to overcome barriers related to broad scale implementation of technology in health systems. We aimed to develop a process guide for eScreening implementation in VHA clinics to automate self-report screening of mental health symptoms and psychosocial challenges.

**Methods:**

This was a two-phase, mixed methods implementation project building on an adapted quality improvement method. In phase one, we adapted and conducted an RPIW to develop a generalizable process guide for eScreening implementation (eScreening Playbook). In phase two, we integrated the eScreening Playbook and RPIW with additional strategies of training and facilitation to create a multicomponent implementation strategy (MCIS) for eScreening. We then piloted the MCIS in two VHA sites. Quantitative eScreening pre-implementation survey data and qualitative implementation process “mini interviews” were collected from individuals at each of the two sites who participated in the implementation process. Survey data were characterized using descriptive statistics, and interview data were independently coded using a rapid qualitative analytic approach.

**Results:**

Pilot data showed overall satisfaction and usefulness of our MCIS approach and identified some challenges, solutions, and potential adaptations across sites. Both sites used the components of the MCIS, but site 2 elected not to include the RPIW. Survey data revealed positive responses related to eScreening from staff at both sites. Interview data exposed implementation challenges related to the technology, support, and education at both sites. Workflow and staffing resource challenges were only reported by site 2.

**Conclusions:**

Our use of RPIW and other QI methods to both develop a playbook and an implementation strategy for eScreening has created a testable implementation process to employ automated, patient-facing assessment. The efficient collection and communication of patient information have the potential to greatly improve access to and quality of healthcare.

**Supplementary Information:**

The online version contains supplementary material available at 10.1186/s43058-021-00132-x.

Contributions to the literature

- These findings show how quality improvement methods can be used to both develop a playbook and an implementation strategy for health technologies like eScreening.
- A Rapid Process Improvement Workshop can be an important factor in the adoption of health technology, but organizational factors also need to be addressed.
- Through our experience implementing eScreening, we have found that successful adoption of health technology needs to be flexible and contain multiple components.
- Our use of quality improvement methods and strategies have created a testable implementation process to employ self-report health screening technology.

## Background

The Veterans Health Administration (VHA) has emphasized technology modernization to improve the provision of health services to the nearly 9 million veterans it serves [1]. Given strong feasibility evidence of technology to effectively automate self-report screening in a variety of health settings [2–5] and studies that have shown the benefits of electronic self-report screening for patients, providers, and health systems [6–10], the VHA developed a comprehensive mobile screening technology (eScreening) that provides customized and automated self-report mental and physical health screening via mobile tablet for veterans seen in VHA healthcare settings [11].

eScreening is a web-based application that was developed with user-centered-design methodology [12, 13] from patient and provider users’ feedback resulting in high veteran satisfaction scores [14]. eScreening reads from and writes to the VHA electronic medical record (EMR) allowing for customized screening and feedback for veterans, real-time alerts for clinicians, and seamless EMR data integration. A pilot of eScreening compared to paper screening in a sample of 1372 newly enrolling post-9/11 veterans found that eScreening improved rates and speed of screening completion, referrals to needed care, and completion of suicide risk assessments when indicated [11]. eScreening results were comparable to evaluations of other electronic self-report screening programs [2–5], and it was subsequently named a Gold Standard Promising Practice for Diffusion throughout VHA [15, 16].

There is agreement about the value and potential impact of health technology, but limited knowledge of how best to broadly implement and scale up health technologies [17]. Potential implementation barriers of digital health interventions within healthcare settings can include a range of organizational and staff-related factors, such as perceptions regarding user motivation and lack of staff training to use digital devices/tools/systems/platforms [18]. Key strategies for a successful implementation of health technology include planning, training and assessment of staff, and continuous evaluation and monitoring [18]. Similar facilitators were identified in an initial evaluation of eScreening implementation in four VHA settings [19]. Other technology implementation factors include characteristics of the intervention (e.g., its cost, complexity, and adaptability), the characteristics of the staff, and support for the digital interventions [18].

Quality improvement methods and strategies employed in health care may offer solutions to overcome barriers related to broad scale implementation of technology in health systems [17]. One example is a Lean Six Sigma Rapid Process Improvement Workshop (RPIW). A RPIW is a highly detailed intervention in which preliminary information on current practice is collected prior to, and then systematically analyzed during, a 5-day workshop by a group of stakeholders and then used to create a future practice and an action plan that includes measurement and evaluation [20, 21].

The structure and duration of an RPIW can vary to meet the needs of an institution, but the process typically consists of data collection, data analysis, process mapping, factor identification, action planning, and cycles of enactment to overcome barriers [21]. There is a preparatory period of about 6–10 weeks during which waste in the process (i.e., system inefficiencies) is defined, and data is collected to map the current state. The first day of a standard RPIW includes training participants in the RPIW principles and introduces the data gathered in the preparatory phase. The second day consists of collective efforts to further data analysis, such as mapping of a current and future state, conducting a gap analysis, and identifying relevant factors and barriers. The remainder of the week is dedicated to the repetition of action planning, execution, and reevaluation to create the targeted state. Using a Plan-Do-Study-Act framework [22], the plans to achieve the target state are enacted, and reports are completed at 30, 60, and 90 days to evaluate progress.

RPIW components include planning and ongoing measurement, which align well with the known facilitators of successful technology implementation in clinical settings. RPIWs have been used to diminish operational waste; to improve privacy, accuracy of care, and efficiency; to standardize processes; and to decrease wait times in a variety of health care settings [23–26]. RPIWs may also be effective for implementing evidence-based practices in health care settings [21], but there is little research.

We chose RPIW because it is a team-based, performance improvement approach that uses tools, techniques, and philosophies to increase efficiency, improve quality, and reduce variability and because most VHA facilities have the infrastructure and processes in place through the Systems Redesign and Improvement Office [27]. The program is designed to support and facilitate improvement initiatives and develop improvement capacity to reduce variability in care, remove waste, and maximize Veterans’ experience. Each VHA facility has a Systems Redesign and Improvement Coordinator to support these activities [28]. We also selected the RPIW tool because it facilitates collaboration between key stakeholders for quality improvement initiatives focused on the patient’s experience and because it highlights process efficiency [29, 30], which fits well with the pilot results of eScreening. Finally, the RPIW also includes planning and continuous evaluation that has been shown to support implementation of technology [18].

## Methods

This paper follows the guidelines provided in the Revised Standards for Quality Improvement Reporting Excellence (SQUIRE 2.0) to describe the process and findings from a two-phase, mixed methods implementation project. The project started as an adapted quality improvement activity that we undertook in collaboration with the National VHA Office of Patient Care Services. The purpose was to develop a process guide for eScreening implementation in VHA clinics to automate self-report screening of mental health symptoms and psychosocial challenges. In phase one, we adapted and conducted an RPIW to develop a generalizable process guide for eScreening implementation (the eScreening Playbook). In phase two, we integrated the eScreening Playbook and RPIW with additional strategies of training and facilitation to create a multicomponent implementation strategy (MCIS) for eScreening. We then conducted a small pilot evaluation of the feasibility and usefulness of the MCIS at two VHA sites in teams that had previously expressed interest in implementing eScreening.

### Phase one: development of the eScreening Playbook

#### Adapting the RPIW process

**Fig. 1:**
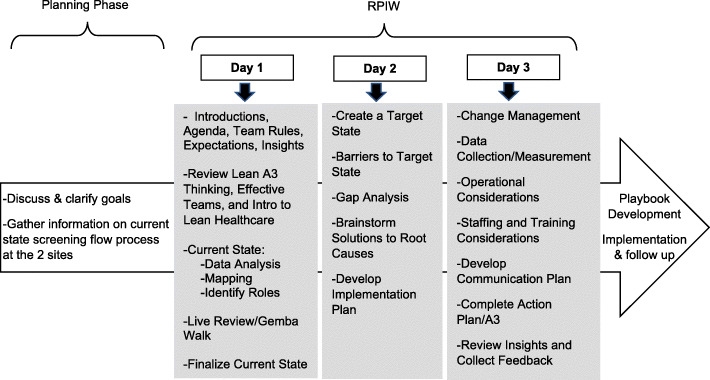
Adapted RPIW process and flow

The key purpose for the adapted RPIW was to develop an eScreening Playbook for use across sites as a starting point for the adoption and implementation of eScreening by healthcare teams. To adapt the RPIW process for this project, we reviewed the key components of a traditional RPIW with a local Lean Six Sigma expert and select stakeholders to identify the components of the original RPIW that needed to be adapted to fit the process with local needs, priorities, and resources. Based on input from our expert and key stakeholder representatives from the National VHA Office of Care Management and Social Work Services and the VHA Transition Care Management Program, we reduced the length of the RPIW process from five to three days to increase feasibility and decrease resources needed. Additional adaptations included a modification in the main outcomes and focus for the process. Unlike a traditional RPIW in which the target state is determined during the process, we set a predetermined target state to include using eScreening as part of the screening process and defined the minimum roles needed to use eScreening. We shifted the focus away from examining current inefficiencies and waste to identifying potential eScreening implementation barriers. We also added a focus on value propositions intended to help teams garner stakeholder support for eScreening implementation. Figure 1 summarizes the adapted RPIW we used.

#### Conducting the adapted RPIW

Next, we chartered a 12-member interdisciplinary workgroup to participate in the adapted RPIW with the goal of developing and piloting an eScreening Playbook. Our local Lean Six Sigma blackbelt level trainer served as the RPIW facilitator. The interdisciplinary RPIW team included social workers, registered nurses, psychologists, innovation specialists, and implementation science researchers. Nearly half the team (*n* =5) were staff from VA San Diego (VASD), but there were representatives from five other VHA sites. Representatives were selected from sites with differing screening processes and patient volume to increase general applicability of the eScreening Playbook. The workgroup began preparing for the RPIW three months in advance by holding bi-weekly telephone meetings to discuss and clarify goals, plan travel arrangements, and gather information about current state screening flow processes at three represented sites. Meetings were facilitated by the VHA eScreening team. All participants were on site at the VASD for the RPIW except for one who participated remotely via video conference.

The agenda for the 3-day RPIW can be found in Additional file 1. During 3 days, the group created team rules, finalized current state maps, defined minimum roles, conducted a live review of the eScreening process at the VASD, developed target state maps, identified barriers and conducted a gap analysis, had presentations on change management and value propositions, and developed implementation and communication plans. The team also identified implementation, operation, and staffing considerations based on lessons learned from the RPIW and previous implementation of eScreening. The RPIW facilitator conducted a brief evaluation of the RPIW with the participants to determine the usefulness and identify strengths and weaknesses of the process. The RPIW included informal and formal presentations, collaborative and facilitated discussions in which the group brainstormed and discussed ideas relevant to the topics. The group used a combination of white board, large and small post-it notes, and PowerPoint to document and collaboratively develop materials for each section (see Additional file 1).

All the RPIW participants (except two who left early due to travel arrangements) provided feedback on the adapted RPIW process. The summary of these evaluations revealed that detailed workflow mapping, full team-member participation, and pre-RPIW prep work, as well as providing breaks throughout the day and using redirection to remain focused on the goal, facilitated the process. Team members identified that the lack of availability of all participants for the entire RPIW and technical issues with the virtual participant were problematic. Finally, the team provided the following lessons learned from the RPIW: video technology for virtual participation is feasible, live visualization the current workflow is critical, and implementation is complex..

#### The eScreening Playbook

Based on the materials from the adapted RPIW process, our team along with the Lean Six Sigma expert, developed the eScreening playbook. Using an iterative approach, we sought input from the participants of the RPIW process for the refinement of the playbook. The complete playbook is available in the supplemental materials (Additional file 2). The key sections of the playbook include a rationale for the implementation of eScreening with a description of the functions, suggestions for overcoming challenges, lessons learned from the field (e.g., comprehensive training for employees), and issues for consideration (e.g., implementation, operational, staffing, and clinical considerations). It also includes considerations for internal preparation, communication, implementation, and data collection and evaluation. An innovative feature of the eScreening Playbook is that it is also designed to be an outline/model for new implementation sites to conduct a site-specific RPIW for eScreening implementation.

### Phase two—implementation pilot

#### Development of a multicomponent implementation strategy

To create a multicomponent implementation strategy (MCIS), we combined the eScreening Playbook and the adapted RPIW developed in phase one with training and facilitation to address the planning, training of staff, and evaluation and monitoring factors posited to facilitate health technology implementation [18].

Our team provided a combination of active and passive training strategies. Active strategies included a 1 h of hands-on training. Passive strategies consisted of access to eScreening video tutorials and the user manual.

Facilitation is a process of interactive problem solving and support that occurs in the context of a recognized need for improvement and within a supportive interpersonal relationship to implement a new intervention or practice [31, 32]. Facilitation provides a mechanism to address factors that impede uptake of an innovation regardless of the source of difficulty such as characteristics of the people, intervention, or the context [33]. Several VHA studies have shown that facilitation improves implementation of complex evidence-based programs [33–35]. Our team provided external facilitation in the form of bi-monthly consultation calls, a site visit at the start of implementation, and technical support as needed.

#### Participating sites

Two pilot sites were selected based on: their interest in implementing eScreening, the availability of technical infrastructure to deploy eScreening software, and the availability of a champion who had permission from their local leadership to implement eScreening into their programs. For both sites, this was the first implementation of eScreening. Site 1 intended to use eScreening as part of the initial health screening for post-9/11 veterans enrolling for healthcare. The team consisted of six social workers and a clinical support assistant. The entire team participated in the implementation process. Site 2 intended to use eScreening as part of ongoing screening and symptom monitoring of veterans receiving posttraumatic stress disorder treatment. The team consisted of five psychiatrists, five psychologists, two social workers, and three affiliated administrative support staff. Only the team lead and two other staff participated in the implementation process.

#### Implementation framework

We selected the Consolidated Framework for Implementation Research (CFIR [36];) as the primary implementation framework for our study because it allows for a multi-level, comprehensive conceptualization of implementation of interventions in real-world settings. CFIR supports the identification of diverse contextual barriers and facilitators of implementation and has been broadly used in the context of the VA. We complemented and expanded CFIR (i.e., Characteristics of Individuals construct) with the Theoretical Domains Framework (TDF [37];), which can facilitate a deeper assessment of the determinants of current and desired behaviors of relevant implementors, (e.g., front-line staff). The Organizational Readiness for Change (ORCA [38];) provided a measurement instrument to identify important Inner Setting characteristics of the sites where eScreening was implemented. CFIR and TDF were primarily used to develop interview guides and survey instruments for this study and to support the analysis of our interviews.

#### Data collection and measures

##### eScreening pre-implementation survey

All individuals that participated in the implementation process at each site were invited to complete the quantitative eScreening questionnaire survey anonymously via Survey Monkey at the start of the implementation. An investigator-created, 29-item eScreening-specific, online survey was used to collect qualitative information from stakeholders. Survey items were derived using constructs from CFIR, TDF, and ORCA.

The survey provided an initial high-level overview of the site staff opinions related to eScreening implementation by efficiently capturing both organizational- and individual-level characteristics that might facilitate or impede implementation. Each item asked respondents to rate agreement to statements such as “The implementation team provided sufficient materials in using and maintaining eScreening quickly” and “For me, using eScreening is worthwhile” on a Likert scale (1–5, strongly disagree to strongly agree; with an option for “Don’t know/not applicable”). The full instrument is available in the supplemental materials (Additional file 3).

##### Implementation process mini interviews

Qualitative data to assess the implementation process for eScreening at each site were collected through 5 open-ended questions used at the bi-weekly telephone facilitation meetings conducted by a member of the SD eScreening team. The questions were designed to identify diverse contextual barriers and facilitators by assessing challenges, solutions, and adaptations [39] to and implementation of various components of the MCIS. Questions included “What are some challenges you encountered regarding the implementation of eScreening at your site over the past 2 weeks?” and “Which components of the implementation strategy did you use during the past 2 weeks?” Data were collected from the implementation site visit to 6 months post-implementation. The full instrument for these “mini interviews” is provided in the supplement files (Additional file 4).

#### Data analysis

We used a complementary mixed-method approach [40] in which qualitative and quantitative data were used to answer different, but related, questions regarding the pilot implementation of eScreening (i.e., quantitative data address organizational- and individual-level characteristics that might facilitate or impede implementation and the qualitative data address eScreening implementation processes). Quantitative eScreening survey data were characterized using descriptive statistics in Excel. To increase cell sizes for analysis, the five response options for each survey question (strongly disagree, disagree, neither agree nor disagree, agree, strongly agree) were collapsed into three categories: disagree, neither agree nor disagree, and agree. The percentage of respondents who endorsed each category was calculated for the 2 sites. The small sample size of survey respondents and the non-normal distribution of the data precluded the use of most statistical techniques for this pilot. As such, the data were descriptively examined for possible trends.

Qualitative data from the bi-weekly facilitation call “mini-interviews” were independently coded by two members of our research team in San Diego using pre-defined codes and using a rapid qualitative analytic approach described by Hamilton and colleagues [41]. CFIR intervention characteristics and process domains were used to support areas for coding including implementation strategies used by each site, challenges of implementation, and adaptations. Coding discrepancies between the reviewers were resolved using a team discussion with the entire VASD research team. The data were summarized and included information pertaining to challenges faced by each site, helpfulness of site visit and calls, and use of playbook.

#### Ethical concerns

This study was reviewed and approved by the VASD Institutional Review Board. Since the project was originally conducted as a quality improvement project, informed consent documents were not required. No PHI was collected. All participants were notified of the project’s purpose and the need to audiotape. Confidentiality agreement and verbal consent was given by each participant.

## Results

### eScreening survey

All invited staff participated in the online eScreening-specific, pre-implementation survey. Seven staff members from site 1 and three staff members from site 2 completed the survey. The findings from the survey are summarized in Table 1 (Additional file 5). For site 1, all staff agreed with 23 (49%) and disagreed with one (2%), and staff were mixed on 23 (49%) of the remaining items. For site 2, 100% of staff agreed with 37 (79%), neither agreed or disagreed with one (2%), disagreed with 1 (2%), and were mixed on eight (17%) of the remaining 47 items. Overall, the opinions of staff members at both sites related to eScreening, its implementation, and their organization were mostly similar and generally positive. For example, all participants from both sites responded identically on 23 of 48 (48%) of the items including rating the strength of eScreening evidence as strong (item 1). Staff respondents from both sites unanimously agreed that eScreening was consistent with clinical practice accepted by VA patients, considered the needs and preferences of VA patients, and had more advantages than disadvantages (items10b-d). Staff universally reported that they were familiar with the content and goals of eScreening, considered using eScreening a responsibility, and had the training and skills to use eScreening (items 11–14). Respondents from both sites also reported that they had sufficient materials, management, and peer support to use eScreening (items 26–28), and that they had a clear plan for using eScreening (item 29).

Some trend differences between the sites were also observed. Site 2 staff had more agreement on statements related to overall leadership support, soliciting opinions of staff about patient care decisions and improving patient education and treatment participation (items 2b and 2c). Site 2 staff also agreed more than site 1 staff on items related to leadership providing/promoting clearly defined roles and responsibilities, team building, and communication (items 5b, 5c, and 5d). More site 1 staff agreed on items related to sufficient support for facilities and staffing (items 7c and 7d). More site 1 staff agreed with statements related to the implementation plan for eScreening, specifically that roles and responsibilities were identified, confidence incorporating eScreening into clinical care, eScreening compatibility with work routine, and having necessary resources for eScreening (items 8a, 8b, 15, 22, and 25).

### Implementation process mini interviews

Findings from the qualitative analysis of implementation process “mini interviews” on bi-weekly facilitation calls are summarized by key areas of interests of use of MCIS components, challenges, and adaptations.

The components of the MCIS were used with slight variation across the two VHA pilot sites. The site 1 team reported less use of the playbook and requested assistance with most technical challenges, stating, “It’s more valuable to have a person to talk to who can resolve issues immediately”. “Calling members of the San Diego team” was named as a helpful aspect of the MCIS. The site 2 team reported more use of the eScreening playbook. One staff person said that it was “helpful in terms of trying to build content” for eScreening, but the site also relied on facilitation to address technical challenges. “Having that one person, the go-to person” was noted as a helpful aspect of the MCIS.

Both sites were positive about the site visit. The implementation team facilitated a formal implementation RPIW during the site 1 visit as outlined in the playbook. The entire clinical team participated. One staff reported “I feel like you guys are really thorough and helped us to develop a pretty clear plan”.

The site 2 team decided not to conduct a RPIW to minimize the disruption to patient care by canceling clinics. However, they reported “having the staff here made all the difference.” Another said, “it focused us all and then we were able to …get individual training for folks…and it really addressed a lot of technical issues much more efficiently.”

Facilitation calls from both sites focused primarily on technical issues, which included problems with eScreening software, hardware (iPads or PC) and/or AirWatch connectivity (iPad to secure wi-fi). Staff reported issues with the eScreening software/server citing “glitches in the system, such as clinical reminders not showing up as due in the initial screening batteries and computer jargon/programming data being input into CPRS that shouldn’t be there.” There were several reports of issues related to the hardware used to access eScreening. Staff reported “configuring the iPads was a challenge and that was a big deterrent in implementing it for the whole team.” Staff also reported “experiencing technical difficulties with iPads, only one of several iPads worked.” Related to wi-fi connectivity, staff reported “iPads would go offline.”

Both sites reported the system challenge of obtaining technical support from their local technology (IT) staff. Specifically, one participant stated a challenge was “coordination with IT, when technical difficulties arise.” Another staff reported, “We are always at risk of … having to run a bunch of iPads over to IT”.

A few education/training challenges were also identified by both sites. Staff reported “trouble remembering certain steps when creating eScreening batteries” and “figuring out nuances of eScreening” and “confusion… about certain features.” One staff from site 2 suggested needing “more training for front desk staff” related to their role in eScreening implementation.

Workflow/staffing challenges were reported by site 2 and included difficulty introducing eScreening to staff and being “Short staffed.” For example, staff would “forget to hand out iPads to veterans” or not “care to give them because they are too busy.” Another challenge reported by site 2 related to workflow related to the “different needs for psychiatry and for psychology” staff. Site 2 staff also reported the need for “a lot of investment for the admin staff at the beginning” and reported that a challenge to eScreening implementation was “figuring out how to integrate it into workflow.”

Both sites reported that they had considered making adaptations such as “administering different screening measures” or “adapting certain content to better fit the needs of a specific site,” but neither site had made these adaptations. One staff person from site 2 stated, “So, we haven’t made any alterations yet because we really haven’t gotten to where we were trying to get yet.”

## Discussion

We described the adaptation of an RPIW to develop an eScreening playbook and the subsequent development and pilot of a MCIS that included the eScreening playbook and RPIW, training, and facilitation. Our team implemented eScreening in two VHA sites using these strategies. Pilot data showed overall satisfaction and usefulness of our approach and identified some challenges, solutions, and potential adaptations across the sites. Both sites used the components of the MCIS, but site 2 elected not to include the RPIW as part of the process. Both sites’ staff provided positive responses on the quantitative questionnaire related to eScreening, but some slight differential trends emerged. Site 2 reported more leadership support and role communication than site 1, but site 1 had more agreement about the specific roles related to eScreening and its compatibility with workflow and resources than site 2. Both sites reported implementation challenges related to the technology technological support and education; however, only site 2 reported challenges with workflow and staffing resources. Given the RPIW focus on site-specific flow mapping, it is possible that the decision of site 2 to omit the RPIW and rely on the general playbook, which included non-site-specific workflow maps, may have contributed to these challenges.

Our results support prior research that external facilitation is a useful part of an implementation strategy, particularly for more complex programs [33, 36, 37], such as a technology intervention. Like previous findings, our results suggest that external facilitation can be helpful in addressing multiple types of challenges encountered during implementation [33]. Evaluating the usefullness of external facilitation in the context of the cost of the external facilitator may be important to help healthcare systems to determin the relative value of providing that level of support for implementation.

Multiple healthcare institutions have improved the quality of care through the utilization of variations of RPIWs. Sinnott, Breckenridge, Helgerson, & Asch [42] used a RPIW to decrease blood culture contamination rates in the Veteran Affairs Palo Alto Healthcare System. Haugen et al. [43] used a 2-day RPIW to support interdepartmental communication to collaboratively address an issue of facility acquired pressure ulcers. RPIWs have also been used to diminish operational waste and to improve privacy, accuracy of care, efficiency, standardization of processes, and improve wait times [23–26]. Dorflinger et al. [30] used a condensed 2-day RPIW to define and develop interdisciplinary pain clinics that effectively streamlined the consult process, helped develop more effective multimodal treatment plans, and made resources more readily available to aid primary care providers in avoiding common opioid therapy issues in the Veteran Affairs Connecticut Healthcare System.

Our results are consistent with several other projects that applied RPIW and other quality improvement (QI) strategies in the VHA [25, 30, 42, 44] and other healthcare systems [23, 26]. Specifically, all concluded that the application of the RPIW yielded positive results (e.g., improved efficiency, safety, or access to care). However, it is premature to endorse the widespread deployment of RPIW in healthcare systems for several reasons. The extant literature about the use of RPIW in healthcare is small, precluding decisions about its usefulness, and there is very likely a publication bias. Moreover, the goal RPIW is adaptation of a process to a specific context; therefore, comparison across studies is difficult without a standardized methodology. Nonetheless, results from this study, as well as others suggest that RPIW may be a promising method to improve the broad implementation of evidence-based and promising healthcare innovations and increase access, quality, and efficiency of healthcare.

Our results are also consistent with the growing literature supporting use of technology to improve healthcare at the patient, provider, and system level [6–11, 45, 46]. Several studies have shown the feasibility of technology-based self-report health screening automate patient self-report health screening in a variety of health settings [2–5]. Despite the plethora of existing and emerging health technology in the VHA recently reviewed by Haun and colleagues [47], there currently is no widely available patient-facing, mobile technology for self-assessment of mental, medical, and social needs that is integrated with the VHA EMR. eScreening is portable and easy to use and is integrated and synchronized with a secure EMR system (CPRS). These are all features reported by veterans to be important for the success of health technology in the VHA [47]. Future studies are needed to determine if mobile technology for self-assessment results in improved patient outcomes.

Despite the significant need and ample support for technology-based solutions to aid health care delivery, implementation of health technology has been challenging [48–51]. As with most evidence-based processes and treatments, an implementation strategy is paramount. Research on implementation strategies in general is rapidly increasing, and evidence is accumulating to support the use of specific strategies in certain contexts, such as facilitation to implement EBPs in health settings [33, 36, 37]. We gleaned from our experience implementing eScreening that successful adoption of the health technology needs to be flexible and contain multiple components. Hence, we have developed training materials (e.g., user manual and playbook); an adapted QI methodology (RPIW) and playbook; and used external facilitation so that sites could adapt workflow processes to fit specific clinics. Moreover, eScreening itself allows for the tailoring of functions, further increasing flexibility to accommodate different contexts.

Overall, while RPIWs may be a promising method to improve implementation of technology-based practices into healthcare, their use as an implementation strategy has some limitations. There is a considerable investment of resources prior to and during the initial implementation stages. The time needed and potential impact on clinical operations to conduct an RPIW may make this method impractical for some settings. Alternate strategies for gaining input from all stakeholders, such as discussion in team meetings or shared working documents, to ensure appropriate workflow and staffing may be necessary in these cases. Our experience with the deployment of eScreening also underscores the importance of education and training [19]. Despite the development of a playbook for eScreening, our pilot data suggest that resources themselves are not enough to ensure successful implementation; facilitation is also needed. Other researchers have noted that strong leadership is essential for the success of lean tools [52]. In our previous study of the implementation of eScreening [19], we found that not only was leadership endorsement important, but also accountability played a role in the success of implementation. Thus, the use of RPIW can be an important factor in the adoption of technology, but organizational factors also need to be addressed.

While this paper adds to the implementation science literature by describing a systematic method for designing an implementation intervention responsive to key features of context, there are limitations. We did not include patient perspectives in the RPIW process which represent an important perspective in the implementation of patient face interventions. This was a small-scale pilot study with a limited sample size precluding statistical comparisons. We did not operationalize the overarching theoretical model comprehensively, and the model was primarily used to inform data collection instruments and analysis. Similarly, this study only included and assessed a small set of implementation outcomes. Our team is now undertaking a larger scale implementation of eScreening across the VA, and in this newly funded study, we are operationalizing our implementation framework comprehensively and are collecting detailed information about implementation outcomes and context.

## Conclusions

Our use of RPIW and other QI methods to both develop a playbook and an implementation strategy for eScreening has created a testable implementation process to employ automated, patient-facing assessment. The efficient collection and communication of patient information has the potential to greatly improve access to and quality of healthcare. A next step will be to investigate the optimal way to scale up and implement eScreening throughout the VHA to improve mental health services and outcomes for Veterans. We encourage those interested in using a RPIW and/or playbook as an implementation strategy consider evaluating the differential impact of other factors such as training/education, facilitation, and organizational influences such as leadership support on implementation success.

## Supplementary Information

**Additional file 1.** eScreening 3-Day RPIW Agenda.

**Additional file 2.** eScreening Implementation Playbook.

**Additional file 3.** eScreening Implementation Survey.

**Additional file 4.** Biweekly Facilitation Mini Interviews.

**Additional file 5.** Survey Results.

## Data Availability

The datasets supporting the conclusions of this article are available from the corresponding author on reasonable request.

## Abbreviations

EMR: Electronic medical record
EBP: Evidence-based practice
CFIR: Consolidated Framework for Implementation Research
MCIS: Multicomponent implementation strategy
ORCA: Organizational Readiness to Change Assessment
PHI: Personal health information
QI: Quality improvement
RPIW: Rapid Process Improvement Workshop
TDF: Theoretical Domains Framework
VASD: VA San Diego
VHA: Veterans Health Administration
