# Supplementary material for: Adaptation of a quality improvement approach to implement eScreening in VHA healthcare settings: innovative use of the Lean Six Sigma Rapid Process Improvement Workshop
Source: Implement Sci Commun. 2021 Apr 7;2:37. doi: 10.1186/s43058-021-00132-x (PMC8028199; doi:10.1186/s43058-021-00132-x)
Supplement: Supplementary file 1 — Additional file 1. eScreening 3-Day RPIW Agenda. [file 43058_2021_132_MOESM1_ESM.docx]

| Day One | | |  |
| --- | --- | --- | --- |
| **Time** | **Activity** | **Methods** | **Who** |
| 0830 | RPIW Kick-Off | Informal review of purpose/goal of RPIW | Facilitator |
| 0840 | Introductions/Icebreaker | Sequential introductions with discipline, role, hobby and an interesting self-fact | All Participants |
| 0900 | Review Agenda, Team Rules, Expectations, Insights, Parking Lot (to list topics out of scope), and Quote of the Day (for inspiration) | Informal description and discussion with multiple 25” x 30” post-it notes to document | Facilitator and all participants |
| 0915 | Review Lean A3 Thinking, Effective Teams, and Intro to Lean Healthcare | Formal PowerPoint Lecture | Facilitator |
| 0945 | Break |  |  |
| 1000 | Current State - Data Analysis | Collaborative discussion documented on white board | All participants |
| 1030 | Current State - Mapping | Facilitated discussion: identify steps in current process and placed on board with 3” x 3” post-it notes on whiteboard | Facilitator and all participants |
| 1130 | Current State – Identify Minimum Roles | Collaborative discussion: identify all roles involved in the current state to create swim lanes (Playbook slide 13) | Facilitator and all participants |
| 1200 | Lunch |  |  |
| 1300 | Prepare for Gemba | Informal presentation: meaning and value of Gemba | Facilitator |
| 1330 | Live Review/Gemba Walk | Observe and speak to providers in the clinical setting | All participants |
| 1500 | Finalize Current State | Collaborative discussion review/discussion | Facilitator and all participants |
| 1530 | Review Team Rules, Insights, and Next Day’s agenda | Facilitated discussion / informal presentation | Facilitator and all participants |
| Day Two | | |  |
| 0830 | Review Agenda and Quote of the Day | Informal presentation | Facilitator and one participant |
| 0840 | Create a Target State | Facilitated discussion: add steps (Playbook slide 16) with 3” by 3” post-it notes and re-arrange map on white board | Facilitator and all participants |
| 0945 | Break |  |  |
| 1000 | Barriers to Target State | Facilitated discussion: brainstorm barriers on white board | Facilitator and all participants |
| 1030 | Gap Analysis | Facilitated discussion: identify causes related to identified barriers on white board (Playbook slide 22) | Facilitator and all participants |
| 1100 | Brainstorm Solutions to Root Causes | Facilitated discussion: identify solutions on white board (Playbook slide 23) | Facilitator and all participants |
| 1200 | Lunch |  |  |
| 1300 | Develop Implementation Plan | Facilitated discussion: identify what steps are needed, who will complete, and by when (Playbook slide 25) | Facilitator and all participants |
| 1445 | Break |  |  |
| 1530 | Review Team Rules, Insights, and Next Day’s agenda | Facilitated discussion / informal presentation | Facilitator and all participants |
| Day Three | | |  |
| 0830 | Review Agenda and Quote of the Day | Informal presentation | Facilitator and one participant |
| 0840 | Group Photo |  |  |
| 0900 | Change Management | Formal PowerPoint Lecture and Collaborative discussion | Facilitator and all participants |
| 0945 | Break |  |  |
| 1000 | Data Collection/Measurement | Facilitated discussion: identify potential metrics and data sources related to implementation (Playbook slide 32) on white board | Facilitator and all participants |
| 1030 | Operational Considerations (Playbook slide 25) | Facilitated discussion: identify impact of implementation on facility operations (Playbook slide 36) on white board | Facilitator and all participants |
| 1130 | Staffing and Training Considerations | Facilitated discussion: identify impact of implementation on facility operations (Playbook slides 31 and 37) on white board | Facilitator and all participants |
| 1200 | Lunch |  |  |
| 1300 | Develop Communication Plan | Facilitated discussion: identify impact of implementation on facility operations (Playbook slides 26, and 28-30) on white board | Facilitator and all participants |
| 1430 | Break |  |  |
| 1445 | Review Team Rules, Insights, and Conclude RPIW | Facilitated discussion / informal presentation | Facilitator and all participants |
