# Supplementary material for: Adaptation of a quality improvement approach to implement eScreening in VHA healthcare settings: innovative use of the Lean Six Sigma Rapid Process Improvement Workshop
Source: Implement Sci Commun. 2021 Apr 7;2:37. doi: 10.1186/s43058-021-00132-x (PMC8028199; doi:10.1186/s43058-021-00132-x)
Supplement: Supplementary file 2 — Additional file 2. eScreening Implementation Playbook. [file 43058_2021_132_MOESM2_ESM.pdf]

VA Integrated Case Management

Coordination Collaboration Communication

eScreening Implementation: Playbook

September, 2016

VA Center of Excellence CESAMH Stress and Mental Health

Veterans Health Administration

myVA Putting Veterans First

VA Nursing

VA Care Management and Social Work Services
Care and Compassion across the Continuum

Table of Contents

Rational for eScreening	4
Overcoming Current Challenges with eScreening	5
Why Do We Need an eScreening Implementation Playbook?	6
Lessons Learned From the Field	7
Step for Implementation Success	8
Prepare: RPIW & A3	9-10
Process Mapping	11
Mapping the Current State	12-15
Mapping the Target State	16-20
Implementation Planning	21
Conduct a Gap Analysis	22
Countermeasure/Solution Approach	23
Collaborate	24
Implementation Plan Template	25
Communicate Internally	26
Lead Change	27
Communication Plan-Template	28
Value Proposition and Message	29
Communicate To Veterans	30
Comprehensive Training for Employees	31
Collect and Evaluate Data	32

VA Integrated Case Management
Coordination Collaboration Communication

Table of Contents (cont.)

Implementation Considerations	33
General Considerations	34-35
Operational Considerations	36
Staffing Considerations	37
Clinical Considerations	38

Rational for eScreening

eScreening is a software application that automates the manual processes used for screening Veterans in VA healthcare settings for health issues. eScreening has the following functions:

- Two-way VistA/CPRS communication, which assigns needed health screens and submits the information to CPRS to satisfy clinical reminders and generate a clinical note for review and signature
- Real-time scoring of screens for staff notification of high-risk Veterans for same-day care, and generates a personalized feedback sheet for the Veteran
- Dynamic progress reporting allows staff and Veterans to monitor health symptoms over time. A robust forms editor allows the system to be flexible to fit any clinic, as it can edit nearly every aspect of the system
- Harnesses the power of technology to improve the Veteran's experiences in VHA clinics and allow clinicians to practice at the top of their licenses

View a demonstration of the system here: https://www.vapulse.net/videos/6943

Overcoming Current Challenges with eScreening

VHA serves about 9 million Veterans each year, with an estimated annual 8-12% increase. More than 1.6 million troops have been deployed to conflicts in the middle east, needing healthcare upon discharge. Efficient and evidence-based screening, assessment, and outcome monitoring are critical to ensure VHA care meets Veterans' needs, especially in Transition Care Management (TCM). eScreening has these critical capabilities to overcome challenges including:

- Allows patient-directed reporting of health symptoms and psychosocial concerns with immediate patient feedback and results documented to CPRS
- Satisfies clinical reminders and generates a clinical note for review
- Enables real-time scoring of screens for staff notification of high-risk Veterans for same-day care
- Facilitates screening in large-volume clinics AND outcomes monitoring in Mental Health

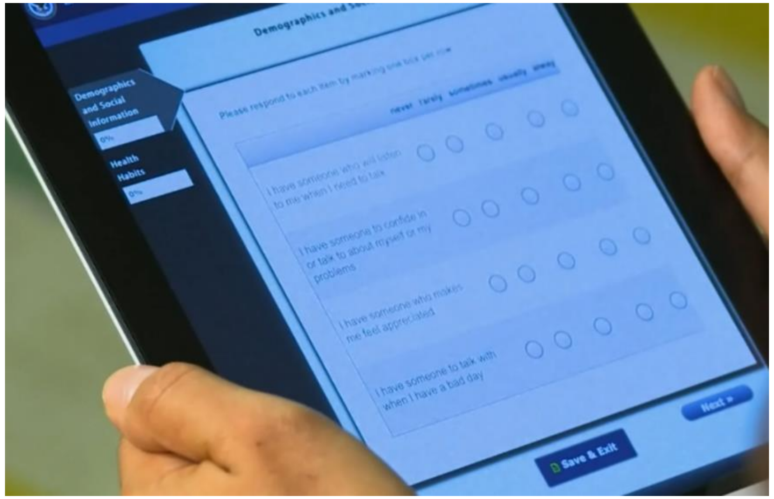

Why Do We Need an eScreening Implementation Playbook?

The Playbook is an idea source for VA facilities that plan to implement or already offer eScreening, as well as to share lessons learned and issues for consideration in the future. The following discussion points should be considered while using the eScreening Implementation Playbook:

- This Playbook is a culmination of the good ideas and best practices shared by the pilot sites
- Pilot programs and lessons learned are intended to be used as the model for an eScreening rollout

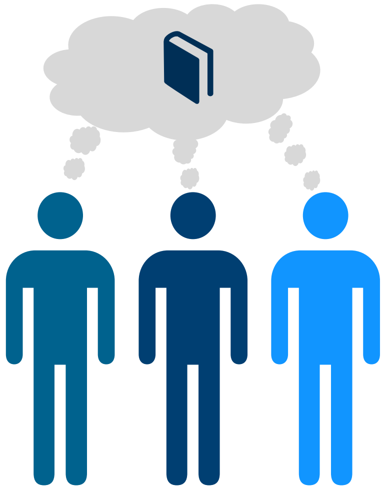

Lessons Learned From the Field

Steps for Implementation Success

Rapid Process Improvement Workshop
- Identifies challenges and solutions unique to each facility
- Facilitates creation of a tailored implementation plan

Internal Preparation
- Modify processes to align with eScreening
- Conduct comprehensive training and designate tasks

Cross-Site Communication
- Have a dialogue with other facilities that have or are in the process of implementing eScreening
- Working together can help ensure preparedness

Communicate the Idea
- Inform the facility about eScreening and its purpose
- Use a variety of communication methods

Implementation
- Determine official start date
- Develop metrics and evaluation plan

Data Collection/Evaluation
- Collect data to evaluate the impact of eScreening
- Maintain communication internally, cross-site, and with Veterans

Prepare: RPIW & A3

The Rapid Process Improvement Workshop (RPIW) process serves to identify current state challenges, the future state solution, and the gaps between them, using the A3 methodology. The A3 process is a helpful tool to organize and visualize the implementation process. Sufficient time should be dedicated for the RPIW and A3 process.

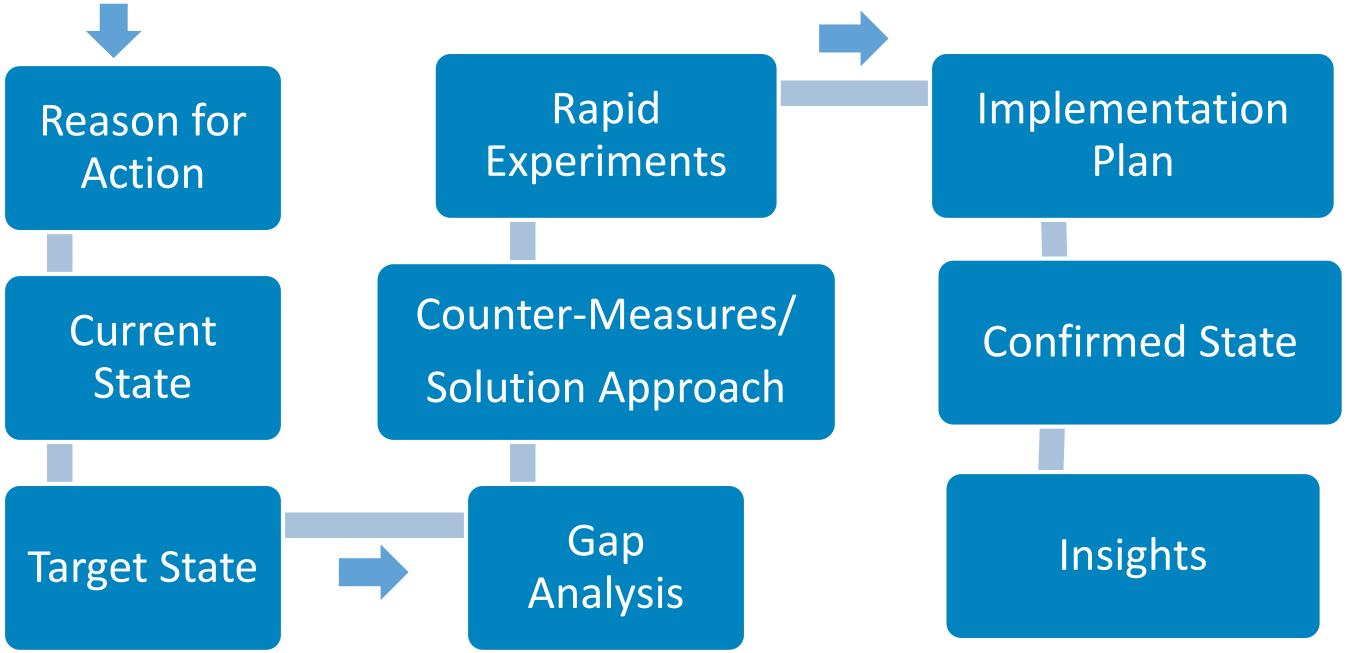

Prepare: RPIW & A3 (cont.)

Proper preparation is essential for a smooth and effective implementation process. Completing the RPIW and A3 outline will help identify tasks that need to be completed prior to implementation. Preparation tasks will be unique to each facility. The below list contains examples and ideas of preparation tasks:

- Internal training
- Internal communication tasks:
  - Develop a communication plan
  - Development and distribution of communications tools such as staff memorandums, intranet postings and staff meetings
- Modify organizational processes to align with the initiative
- Incorporate new procedures
- External communication tasks:
  - Union courtesy notification emails, mailings or postcards for patients, press releases, and other types of public service announcements

Process Mapping

Mapping the Current State

Mapping the current state creates a clear visual representation of the current process. It helps to identify waste and where changes in process can occur. The current state map should be a team effort that is conducted by those people who are involved in the process.

We advise using a flow map with swim lanes, as it easily designates who does what in the process and when do they do it.

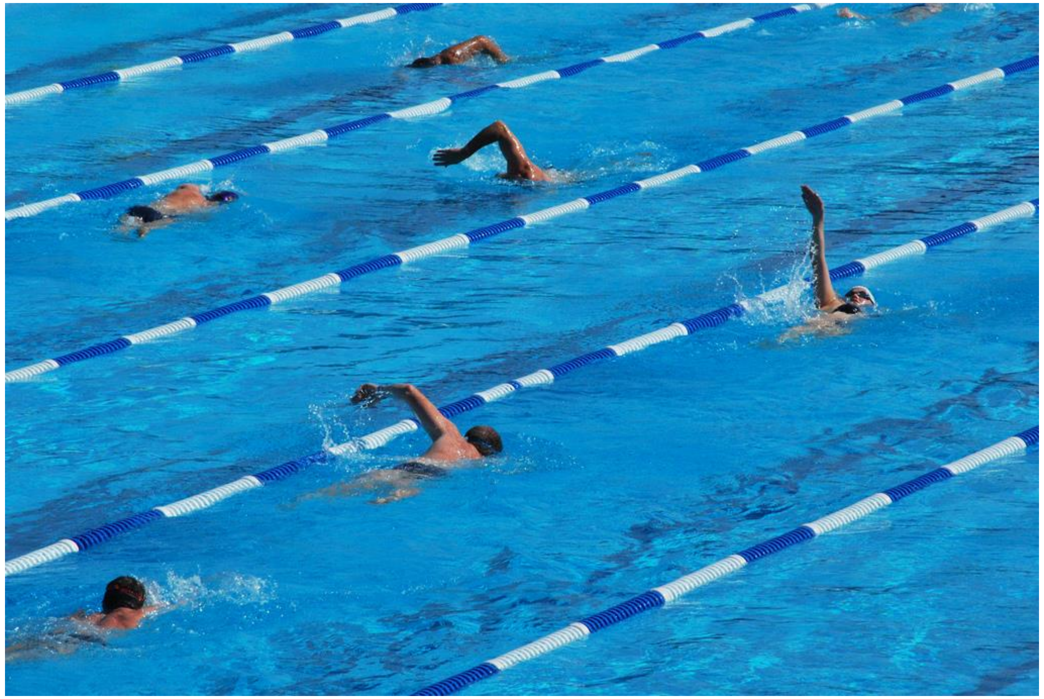

Mapping the Current State Example 1

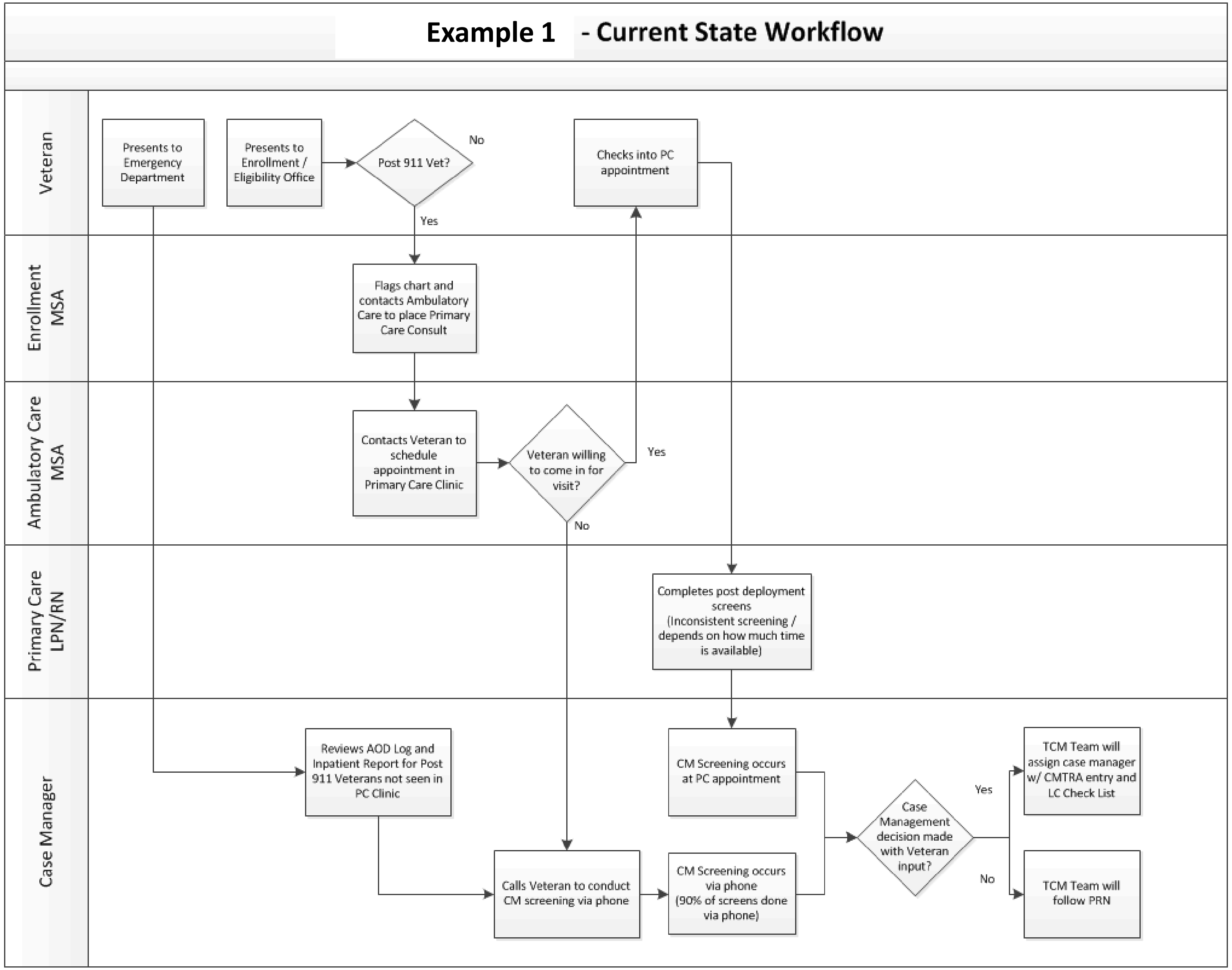

Mapping the Current State Example 2

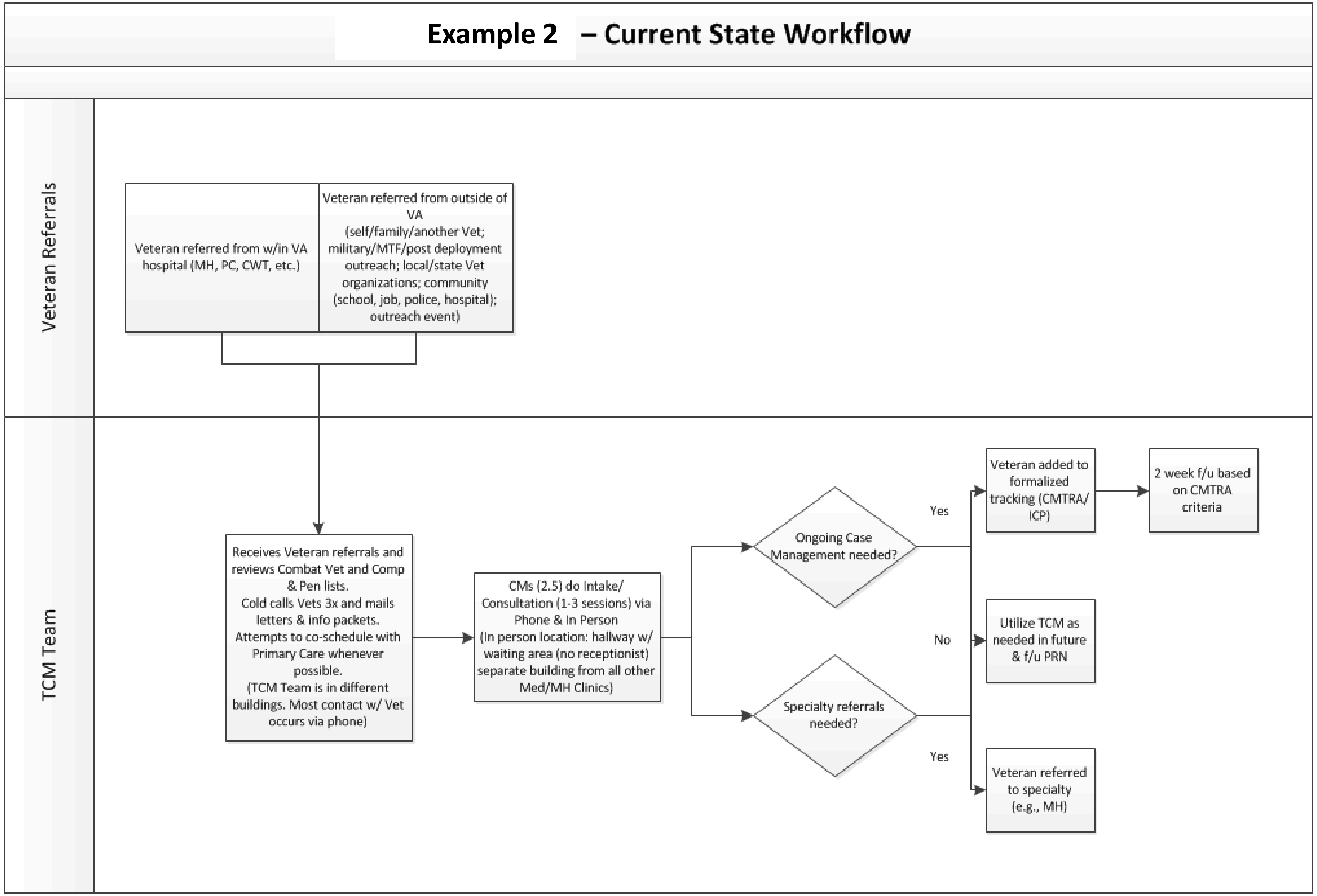

VA Integrated Case Management
Coordination Collaboration Communication

Mapping the Current State Example 3

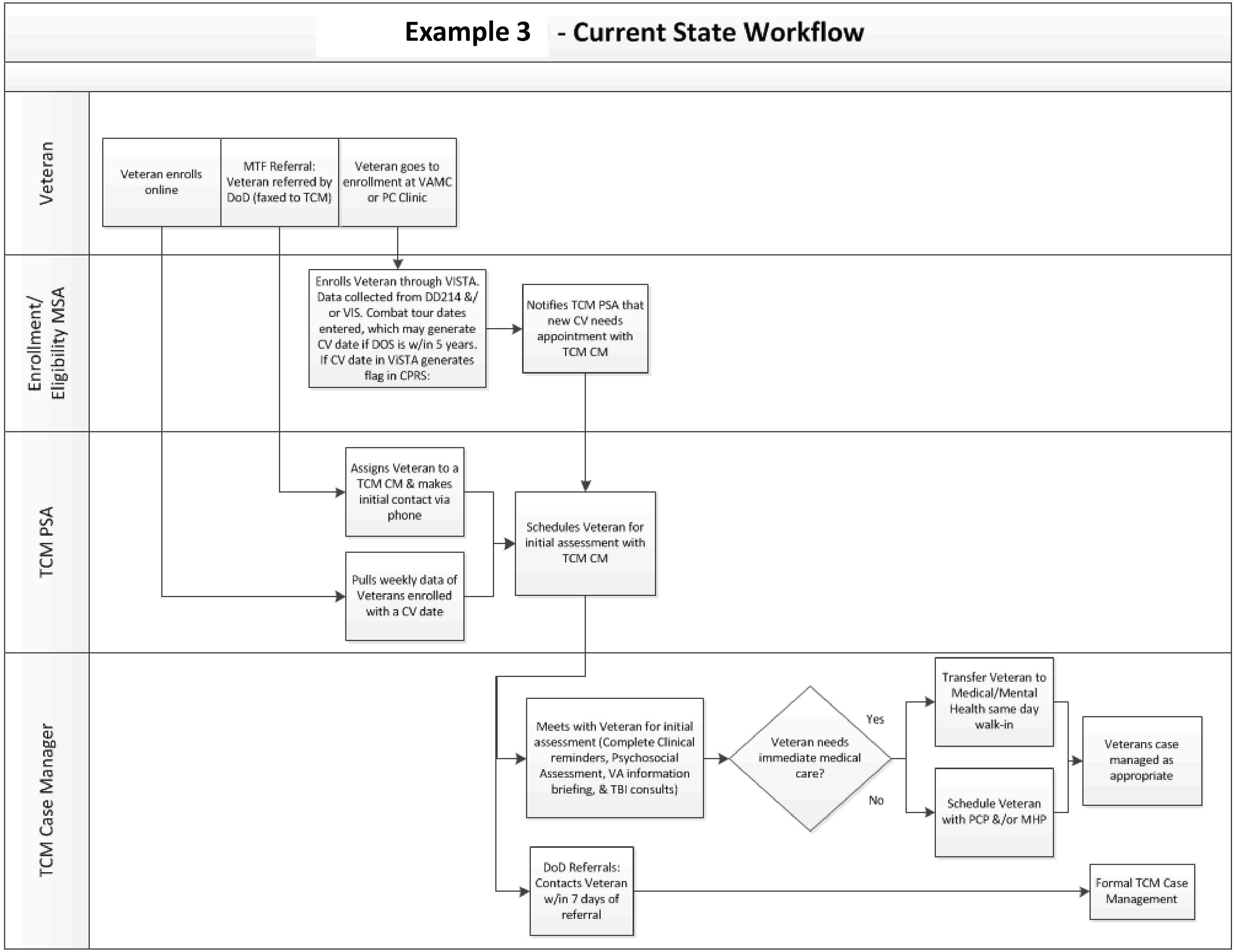

Mapping the Target State

Your target state process map is the ultimate goal of your improvement process and provides you with an objective to work towards. It forms a shared vision of what your improvement process is aiming to achieve. It should be created by the team with guidance from the facilitator and the senior management.

Below are the 8 necessary tasks required to complete an eScreening assessment. These steps should be mapped into your future workflow:

1. Create assessment, including designating the note title, VistA clinic, and assigning the clinician responsible for data collected
2. Hand out tablet
3. Have staff available for Veteran questions (could be staff or volunteer)
4. Have staff to monitor the clinical dashboard
5. Collect, clean, and store tablet when Veteran has completed the eScreening assessment
6. Save information from dashboard to CPRS
7. Provide Veteran with summary printout
8. Clinician evaluation: clinician receives CPRS note and provides care

Mapping the Target State (cont.)

- The following examples have varying degrees of the level of integration. This is based on the number of programs that are involved in the process:
  - High = 3 or more
  - Moderate = at least 2
  - Non-Integrated = 1
- The level of integration can be affected by available resources, the size of the medical center/population, and the physical layout of facilities
- Scheduled appointments and/or the ability to handle walk-ins will also be affected by available resources
- These examples of current and target state maps at individual sites may need to be adapted or modified based on site-specific needs

Target State Example 1

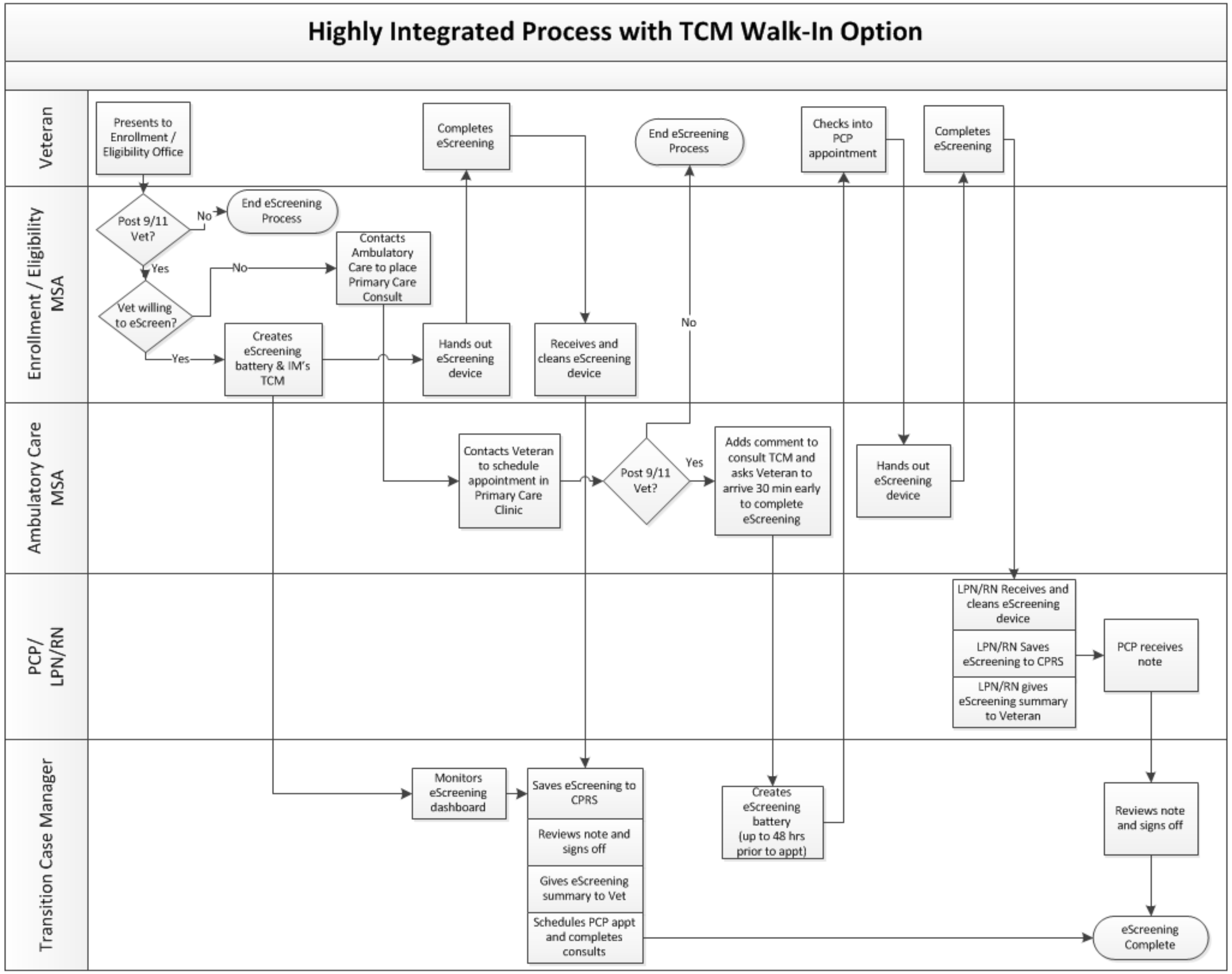

Target State Example 2

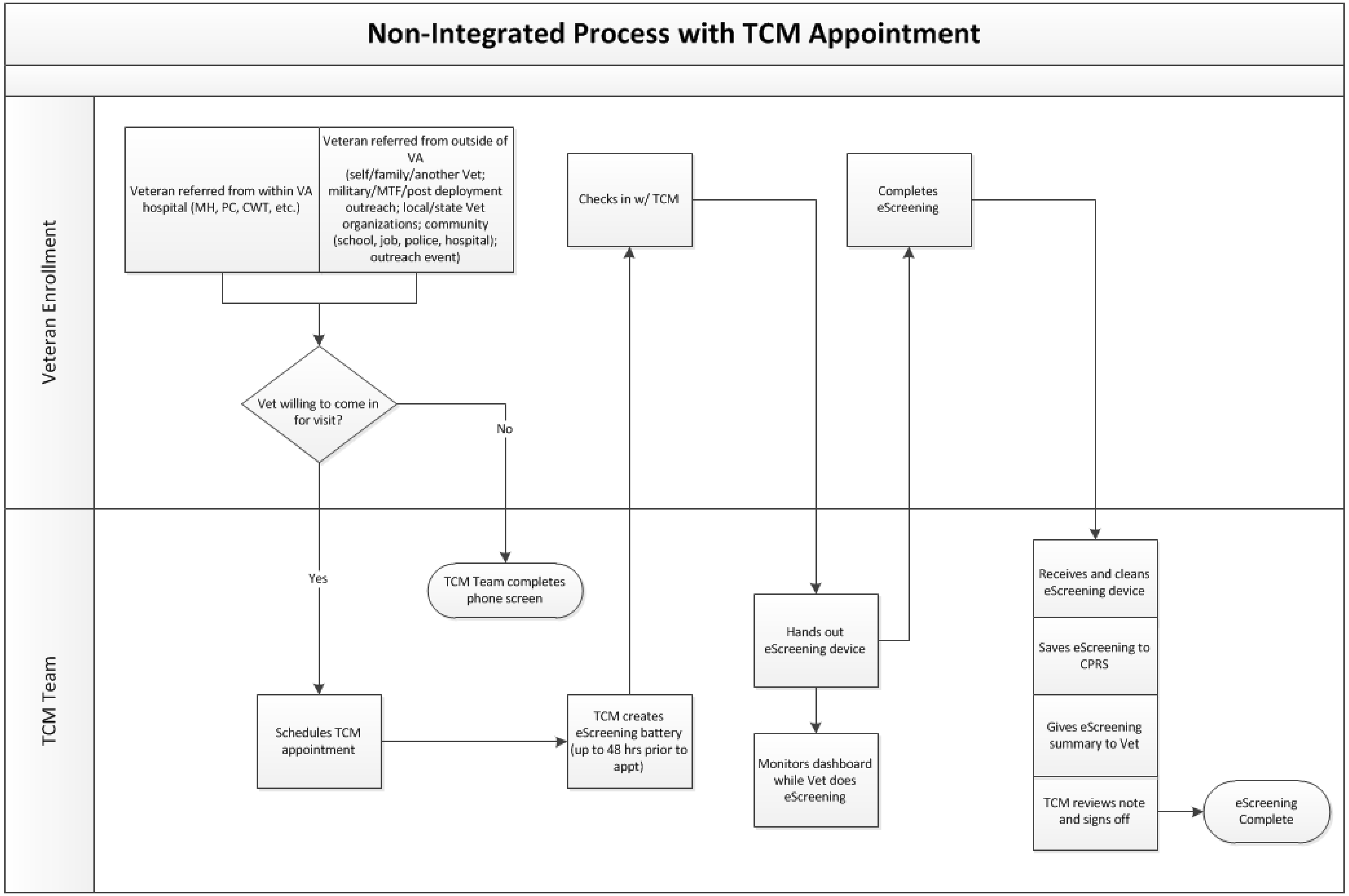

VA Integrated Case Management
Coordination Collaboration Communication

Target State Example 3

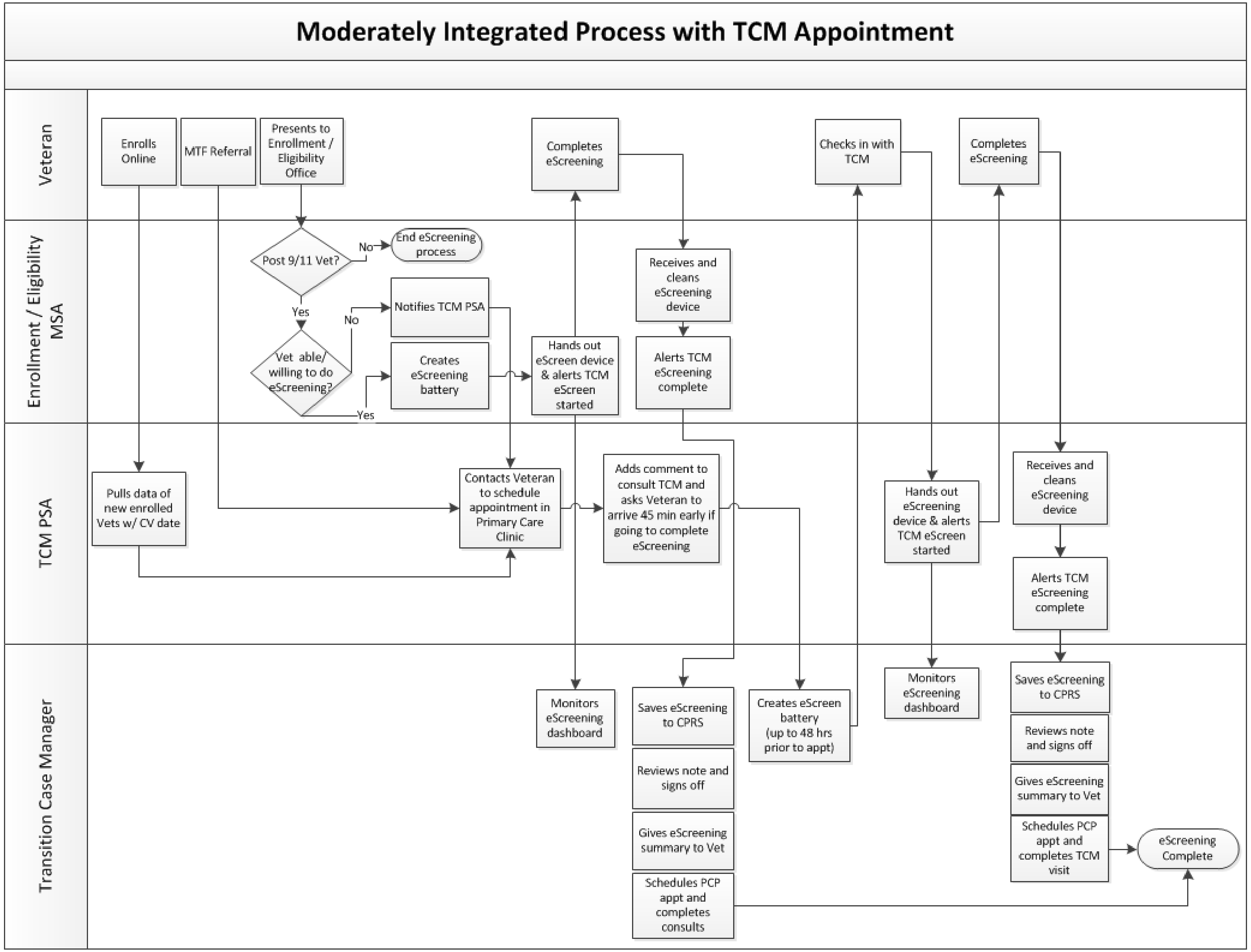

VA Integrated Case Management
Coordination Collaboration Communication

Implementation Planning

Conduct a Gap Analysis

The purpose of the Gap Analysis is to provide the project team a format to do the following:

- Identify potential problems that will arise as a result of the process change
- Consider the root causes of these possible challenges
- Identify focus areas to brainstorm solutions in the next step

Barrier	Direct Cause	Root Cause(s)
Patient late arrival	Parking, traffic, navigating in facility	Not communicating obstacles to Veterans
Not all patients want to use	Don't see the value	Lack of understanding
Patients think it's a survey	Purpose not explained	Lack of education or time for staff
Interferes with front line staff workflow	Patient questions	Not identifying a go-to person
Staff want contingency plan	Staff scared of making mistakes	Lack of autonomy and clarity of responsibilities
Technological issues	Hardware, software, and user error	eScreening has limitations
Resistance to change	Change is difficult	Lack of buy-in and accountability
Physical layout	Space limitations	Veterans not understanding process
Not setting aside time for implementation	Not allocating resources	Not investing resources to change
Patient reluctance to disclose sensitive information	Concerned of confidentiality	Known and unknown consequences

Countermeasure/Solution Approach

The purpose of the Countermeasure/solution approach is to provide the project team a format to do the following:

- Identify solutions based on the gap analysis
- A decision matrix based on impact and effort can be useful in this phase
- Identify your solutions and the problem you anticipate they will resolve

Barrier	If we…	Then we …
Late Arrivals	Inform patient of potential delays at local facility and appointment flow	Prevent late arrivals and have time to do eScreening
	Build eScreening into scheduled appointment time	Have time for eScreening and maintain workflow
Not all patients want to use	Explain to Veterans how eScreening will benefit them	See increase in number of Veterans willing to use eScreening
Technological issues	Have immediate technological expertise	Increase use and staff satisfaction
Resistance to change	Communicate expectations and provide resources	Increase acceptance of eScreening

Collaborate

Cross-Functional Teams

It is critical to engage multiple employee groups who represent a diverse perspective on the challenges and potential solutions for implementing eScreening

Veteran Engagement

Veterans can offer valuable insight during the process of mapping the current state and target state

Cross-Specialty Communication

Often times different service lines have similar issues – collective problem solving can offer innovative solutions

Cross-Site Coordination

The exchange of ideas and experiences between facilities is an efficient method to problem solve and ensure the facility is prepared to implement eScreening. eScreening has a community on VA Pulse at http://vhaindwebsim.v11.med.va.gov/hub2/ppd/practices.html

Implementation Plan Template

An implementation plan is an essential tool designed to illustrate, in detail, the critical steps in developing and starting eScreening. It is a guide that helps staff be proactive rather than reactive and helps maintain accountability and keeps the project on track.

Below is an implementation plan example. There is no standard implementation plan and plans will vary depending on the needs of the facility, but this example can serve as a template and an ideas source for potential action items that may need to be considered.

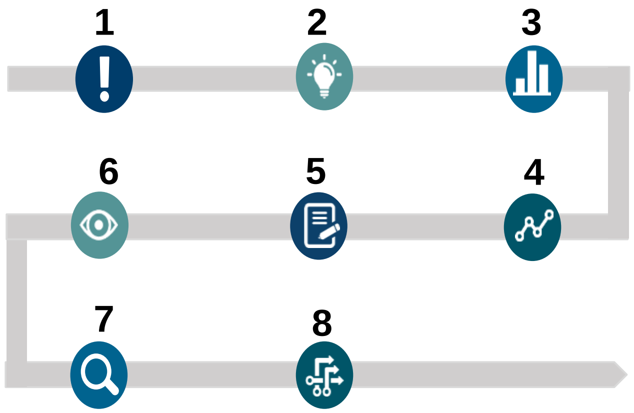

What	Who	When
Identification of Stakeholders	eScreening Champion	October
Rapid Process Improvement Workshop	TCM team and/or other stakeholders	November 1st -3rd
Communication Plan put into action	Public Affairs/Leadership/RPIW members	November 7th-11th
Customization of eScreening Content	Technical Administrator/Clinical Leads	November 14-17th
Training of staff (technical & policies and procedures)	Staff who are mapped into the Target State	November 21-25th
Go-live week	eScreening Stakeholders	November 28th - December 2nd
Feedback & Data analysis meeting	RPIW members/leadership	December 5th
Rapid adjustments to eScreening if needed	RPIW members/leadership	December 6th-December 9th

Communicate Internally

Widespread communications across all levels of the facility will create and maintain awareness of the eScreening initiative and help develop buy-in for the new processes. Consistent communication is essential for sustained engagement and is paramount for successful implementation. Methods to foster consistent communication include:

- Developing a communications plan
- Using a variety of different communication tools such as:
  - Staff memos
  - Intranet postings
  - Emails
  - Staff meetings

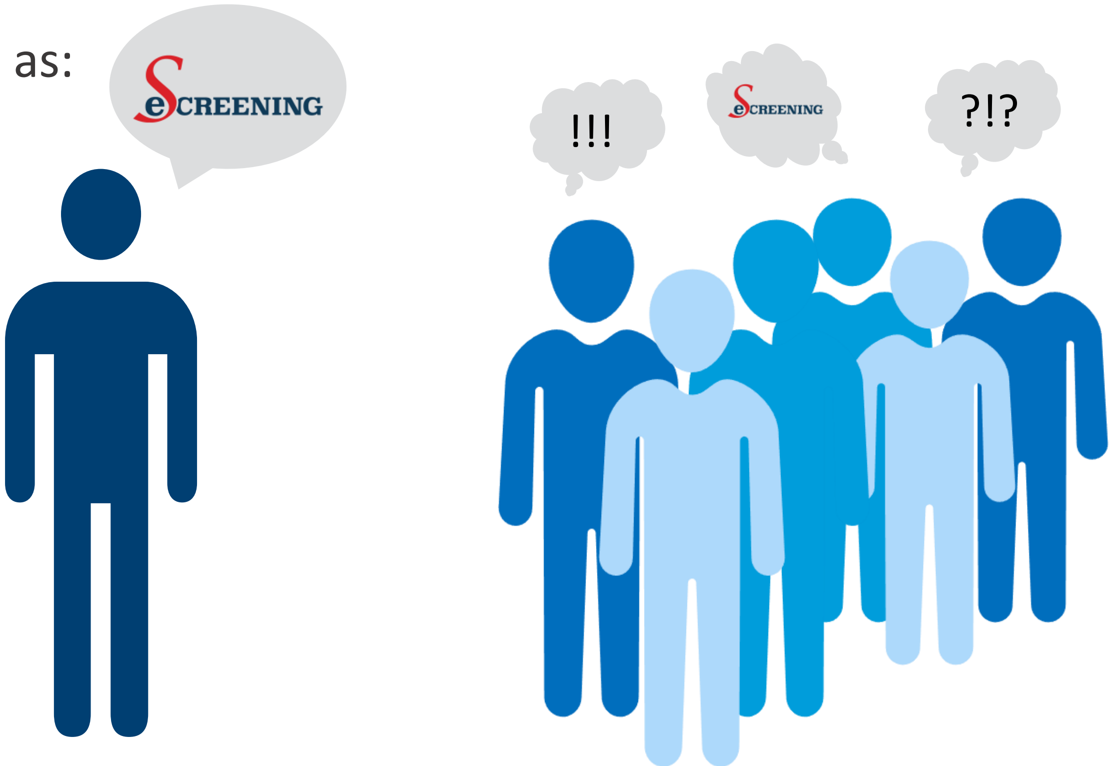

Lead Change

Change is hard. Change Management is a strategy that helps guide how we as facilitators can best support individuals as well as prepare them for successful implementation of a new way of doing things.

Eight Steps to Transforming Your Organization

1. Establish a Sense of Urgency

- Examining market and competitive realities.
- Identifying and discuss crises, potential crises or major opportunities

2. Forming a Powerful Guiding Coalition

- Assembling a group with enough power to lead the change effort
- Encouraging the group to work together as a team

3. Creating a Vision

- Creating a vison to help direct the change effort
- Developing strategies for achieving that vision

4. Communicating the Vision

- Using every vehicle possible to communicate the new vison and strategies
- Teaching new behaviors by the example of the guiding coalition

5. Empowering Others to Act on the Vision

- Getting rid of obstacles to change
- Changing systems or structures that seriously undermine the vision
- Encouraging risk taking and nontraditional ideas, activities and actions.

6. Planning for and Creating the Short Term Wins

- Planning visible performance improvements
- Creating those improvements
- Recognizing and rewarding employees involved in the improvements

7. Consolidating Improvements and Producing Still More Change

- Using increased credibility to change systems, structure and policies that don't fit the vision
- Hiring, promoting and developing employees who can implement the vision
- Reinvigorating the process with new projects, themes, and change agents

8. Institutionalizing New Approaches

- Articulating the connections between the new behaviors and corporate success
- Developing the means to ensure leadership development and succession

Kotter, J. P. (1995). Leading change: Why transformation efforts fail.

Communication Plan-Template

Creating a communication plan will allow for easy tracking of outreach initiatives.

Audience	Group	Mechanism	Who's Responsible	Timing/ Frequency
**Internal**	Clinical Staff	• Email<br>• Staff Meeting		
	PCP	• Email Notification (Initial)- Copy Audiology and Optometry staff Monthly Meeting		
	Nursing	• Monthly Nursing Council Meeting<br>• Weekly Nurse Meeting<br>• Nurse Email Notification		
	Business Office	• BO Email group<br>• Weekly meetings		
	Specialty Services	• Monthly Meeting<br>• Email		
	MSA/ Call Center	• Daily Meeting<br>• Email		
	All Staff	• Medical Staff Meeting<br>• Intranet/webpage<br>• Weekly bulletin (Email)<br>• Message from Director (Email)		
**External**	Patients	• Current patients requesting appointments<br>• VSO/congressional notification<br>• PRNC (for enrolled patients/mail date)<br>• Digital Signs on Kiosks<br>• Electronic bulletin boards (example provided in Toolkit<br>• Press Release		
**Leaders**	VAMC Director	• After morning meeting		
	VACO/ VERC	• Bi-weekly phone calls<br>• Monthly w/ all 3 pilot sites		

VA Integrated Case Management
Coordination Collaboration Communication

Value Proposition and Message

A value proposition is a clear description of what problem eScreening will help solve and how. To encourage implementation, consider what value eScreening offers to each person or group of people that you need to engage. Remember that the perceived value is likely to vary among stakeholders, in part based on their roles and obligations.

Stakeholders	Value Proposition	Message
Administrative Staff	• Satisfied Veterans<br>• Identify Veteran health needs<br>• Help get the best care for the Veteran<br>• Less paperwork	• Way to help Veteran in a way that they couldn't before
Veterans	• Identify immediate needs<br>• Only offer information once<br>• Allows more personalized time with provider<br>• Direct submission to medical record	• Better and faster connection to care you need<br>• More face to face time with provider<br>• Facilitates communication with provider<br>• Individualized feedback<br>• Modernized healthcare
Leaders	• Improved access<br>• Patient centered<br>• Providers work at top of license or scope<br>• Higher completion of clinical reminders<br>• Cost savings<br>• Improved Veterans care	• Meeting performance measures<br>• Better care for Veterans<br>• Helps identify and address needs of high risk patients<br>• Aligns with VA Strategic Plan (see Toolkit)<br>• Improved access<br>• Improved satisfaction and retention<br>• Eliminate waste
Non-TCM Clinicians	• Early identification and intervention<br>• Better quality of referrals<br>• Improved efficiency	• Less duplication<br>• Actionable information that you can use during the visit<br>• Reduced documentation burden<br>• Offers useful baseline information
TCM Case Managers	• Encourages disclosure<br>• Direct submission to medical record<br>• Meet performance measures<br>• Decrease time documenting<br>• Not having to score assessment<br>• Efficient collection of comprehensive information<br>• Increase screens per month<br>• Useful in completing LC checklist<br>• Triage assistance	• Less administrative work and more clinical time<br>• Less time cold calling<br>• Less time documenting<br>• Less duplication<br>• Helps understand Veteran priorities<br>• Meet measures<br>• Helps prioritize which Veterans to see and when<br>• Helps identify who needs care management services
OI&T	• Help get the best care for the Veteran<br>• Be part of innovation	• Way to help Veteran in a way that they couldn't before<br>• Help modernize healthcare

Communicate To Veterans

Robust marketing of this service to Veterans is a critical priority. Facilities need to clearly communicate to Veterans about the availability of eScreening and how they can take advantage of it. Creating a communication plan will allow for easy tracking of outreach initiatives. Below are some potential communication channels:

- Clinicians and schedulers
- Upon check-out from front desk staff
- Electronic messages on displays around the facility
- Mailings (post cards)
- Message boards/forums
- Involvement of Public Affairs in communication strategy
- Social media (Twitter, Facebook)
- My HealtheVet

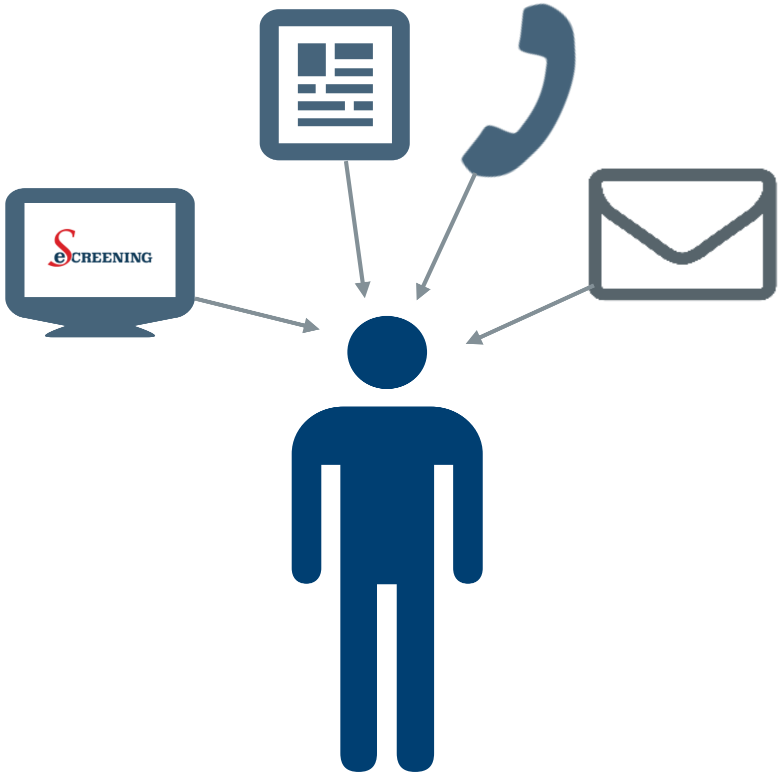

Comprehensive Training for Employees

In order to effectively and accurately implement the eScreening, all applicable employees must receive comprehensive training. Having dedicated time set aside for training is essential to the successful implementation of eScreening.

- Role based training for administrative, clinical, technical staff, and volunteers (see toolkit)
- Facility should identify local training coordinator and implementation manager (see toolkit)
- Training support may be available from eScreening developers (Center of Excellence for Stress and Mental Health)
- Establish local training for new staff and volunteers

Collect and Evaluate Data

Although each facility may elect to track different or additional metrics, the below set of data points proved very useful in evaluating the impact of eScreening.

Metric	Data Source
Patient Satisfaction	• eScreening
Staff Satisfaction	• Manual collection
% of Clinical Reminders Cleared	• VHA Support Service Center (VSSC) website (see Toolkit)
% of Assessments Completed/Created	• eScreening
% of CMSA Domains Captured	• eScreening<br>• Manual collection
% of Veterans at High Risk	• eScreening

Implementation Considerations

General Considerations

Before the official kickoff date, several key conditions should be met to eliminate potential pitfalls. The following are some key examples and considerations: Ensure that all staff are educated on eScreening, all necessary preparations have been completed, the initiative has adequate support and resources, and the operational processes are aligned to accommodate eScreening.

Size matters

VA facility size and patient volume impacts implementation and coordination, and communication is critical especially at large VA facility sites to ensure a smooth transition

Time is critical

Although it was feasible to stand up eScreening within 1 month of completing the RPIWs, it would have been better to allow more time to ensure that all levels of staff were comfortable with the transition and how it may specifically impact their individual job duties

Manage up

Since there is the potential that eScreening may change clinical flows, it is critical to keep medical center leadership informed and supported during the initial stages of the pilot

General Considerations (cont.)

Pilot sites shared many insightful considerations for eScreening implementation.

- eScreening aligns with VA Strategic Plan (see Toolkit)
- Not all facilities can have eScreening due to OI&T limitations
- There are hard costs associated with establishing eScreening technology and soft costs of allocating staff to support implementation
- There are costs associated with sustainment
- Consider collecting Pre and Post implementation data. Consultation available through VA Center of Excellence for Stress and Mental Health (CESAMH) at SDCVAMCCESAMHeScreening@va.gov
- This is the only tool that performs certain functions in the VA and public sector
- Originated by CESAMH at the VA San Diego Healthcare System

Operational Considerations

With any performance improvement initiative, operations will be affected. eScreening is no different. The pilot sites reported similar impacts to their facility operations after implementation.

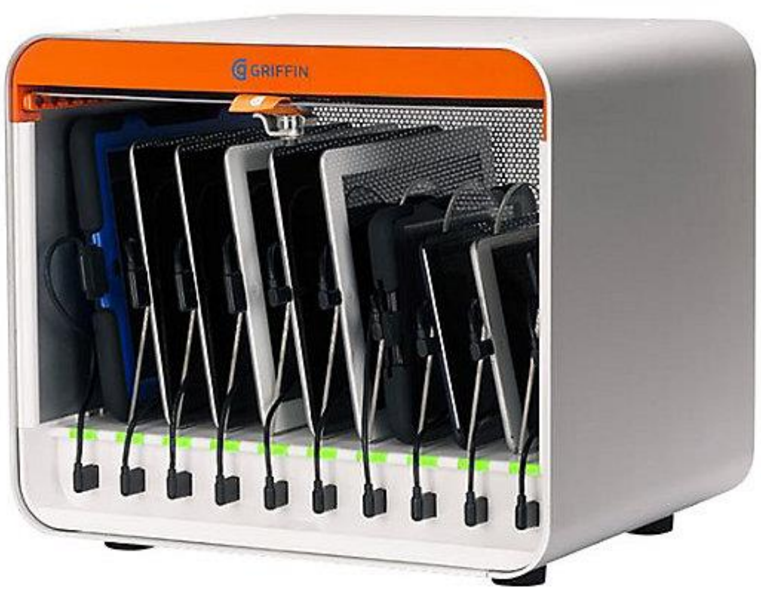

Operational Considerations

- Increased knowledge of demand for specialty services
- Increased need for same-day assessments or scheduling
- Potential impact on OI&T servers
- Allocation of secured space for storage and maintenance of devices
- Need printer access for Veteran Screening Summary

Staffing Considerations

To the best of their ability, facilities need to project or predict demand changes and assess staffing needs prior to implementation. Additional staff may be needed, especially front end staff.

Staffing

- OI&T support
- Potential utilization of volunteers to assist eScreening process
- Allocation of staff time (administrative/clinical) for monitoring the process
- Staff time for training and implementation

Clinical Considerations

eScreening makes a positive impact on Veterans' care, according to all Transition Team focus group participants, because the rapid turnaround of information means Veterans who need immediate care – those who may be suicidal or homicidal, for example – can get the attention and care they need on the same day.

However, when introducing this new technology, three key clinical considerations are:

- The potential for increased identification of positive screens may require additional clinical follow up
- Implementation may require adaptation of staff roles
- Auto-generated notes still need to be reviewed for accuracy prior to signing

THANK YOU
