# Supplementary material for: Adaptation of a quality improvement approach to implement eScreening in VHA healthcare settings: innovative use of the Lean Six Sigma Rapid Process Improvement Workshop
Source: Implement Sci Commun. 2021 Apr 7;2:37. doi: 10.1186/s43058-021-00132-x (PMC8028199; doi:10.1186/s43058-021-00132-x)
Supplement: Supplementary file 3 — Additional file 3. eScreening Implementation Survey. [file 43058_2021_132_MOESM3_ESM.pdf]

eScreening Implementation Survey

VA Site:
Number of Years:

1. Please rate the strength of eScreening in your opinion, on a scale of 1 to 5 where 1 is very weak and 5 is very strong evidence:	Very weak	Weak	Neither weak nor strong	Strong	Very Strong	Don't know/not applicable
	1	2	3	4	5	

For each of the following statements, please rate the strength of your agreement with the statement, from 1 (strongly disagree) to 5 (strongly agree).

2. Senior leadership/clinical management in your organization:	Very weak	Weak	Neither weak nor strong	Strong	Very Strong	Don't know/not applicable
a. Rewards clinical innovation and creativity to improve patient care	1	2	3	4	5	
b. Solicit opinions of clinical staff regarding decisions about patient care	1	2	3	4	5	
c. Seek ways to improve patient education and increase patient participation in treatment	1	2	3	4	5	

3. The proposed practice changes with eScreening should be effective, based on current scientific knowledge	Very weak	Weak	Neither weak nor strong	Strong	Very Strong	Don't know/not applicable
	1	2	3	4	5	

4. Staff members in your organization:	Very weak	Weak	Neither weak nor strong	Strong	Very Strong	Don't know/not applicable
a. Have a sense of personal responsibility for improving patient care outcomes	1	2	3	4	5	
b. Cooperate to maintain and improve effectiveness of patient care	1	2	3	4	5	
c. Are willing to innovate and/or experiment to improve clinical procedures	1	2	3	4	5	
d. Are receptive to change in clinical processes	1	2	3	4	5	

5. Senior leadership/Clinical management in your organization:	Very weak	Weak	Neither weak nor strong	Strong	Very Strong	Don't know/not applicable
a. Provide effective management for continuous improvement of patient care	1	2	3	4	5	
b. Clearly define areas of responsibility and authority for clinical managers and staff	1	2	3	4	5	
c. Promote team building to solve clinical care problems	1	2	3	4	5	
d. Promote communication among clinical services and units	1	2	3	4	5	

6. Senior leadership/clinical management in your organization:

	Strongly disagree	Disagree	Neither agree nor disagree	Agree	Strongly agree	Don't know/not applicable
a. Establish clear goals for patient care processes and outcomes	1	2	3	4	5	
b. Provide staff members with feedback/data on effects of clinical decisions	1	2	3	4	5	
c. Hold staff members accountable for achieving results	1	2	3	4	5	

7. In general, and within my unit/team, when there is agreement that change needs to happen:

	Strongly disagree	Disagree	Neither agree nor disagree	Agree	Strongly agree	Don't know/not applicable
a. We have the necessary support in terms of financial resources	1	2	3	4	5	
b. We have the necessary support in terms of training	1	2	3	4	5	
c. We have the necessary support in terms of facilities	1	2	3	4	5	
d. We have the necessary support in terms of staffing	1	2	3	4	5	

8. The implementation plan for eScreening:	Strongly disagree	Disagree	Neither agree nor disagree	Agree	Strongly agree	Don't know/not applicable
a. Identified specific roles and responsibilities	1	2	3	4	5	
b. Clearly describe tasks and timelines	1	2	3	4	5	
c. Includes appropriate provider/patient education	1	2	3	4	5	
d. Acknowledges staff input and opinions	1	2	3	4	5	

	Strongly disagree	Disagree	Neither agree nor disagree	Agree	Strongly agree	Don't know/not applicable
9. The proposed practice changes of eScreening are supported by clinical evidence with VA patients	1	2	3	4	5	

10. The proposed practice changes:	Strongly disagree	Disagree	Neither agree nor disagree	Agree	Strongly agree	Don't know/not applicable
a. Have been well-accepted by VA patients in a pilot study	1	2	3	4	5	
b. Are consistent with clinical practice that have been accepted by VA patients	1	2	3	4	5	
c. Take into consideration the needs and preferences of VA patients	1	2	3	4	5	
d. Appear to have more advantages than disadvantages for VA patients	1	2	3	4	5	

	Strongly disagree	Disagree	Neither agree nor disagree	Agree	Strongly agree	Don't know/not applicable
11. I am familiar with the content and goals of eScreening	1	2	3	4	5	
12. In my work, it is my responsibility to use eScreening	1	2	3	4	5	
13. I have been trained in how to use eScreening	1	2	3	4	5	
14. I have the skills to use eScreening	1	2	3	4	5	
15. I am confident I that I can incorporate eScreening into my clinical care.	1	2	3	4	5	
16. I am confident that I can use eScreening even when others may not	1	2	3	4	5	
17. I am confident I can use eScreening even when there is little time	1	2	3	4	5	
18. For me, using eScreening is useful	1	2	3	4	5	
19. For me, using eScreening is worthwhile	1	2	3	4	5	
20. I intend to use eScreening in the next three months	1	2	3	4	5	
21. I will definitely use eScreening in the next three months	1	2	3	4	5	
22. eScreening is compatible with my work routine	1	2	3	4	5	
23. eScreening has advantages compared to standard care	1	2	3	4	5	

	Strongly disagree	Disagree	Neither agree nor disagree	Agree	Strongly agree	Don't know/not applicable
24. eScreening costs little time to deliver	1	2	3	4	5	
25. Within the organization that I work, all the necessary resources are available to properly use eScreening	1	2	3	4	5	
26. The implementation team provided sufficient materials in using and maintaining eScreening	1	2	3	4	5	
27. I believe that I can count on support from management when things get tough in using eScreening	1	2	3	4	5	
28. I believe I can can count on support from peers who also use eScreening when things get tough	1	2	3	4	5	
29. I have a clear plan how I will use eScreening	1	2	3	4	5	
