# Supplementary material for: Adaptation of a quality improvement approach to implement eScreening in VHA healthcare settings: innovative use of the Lean Six Sigma Rapid Process Improvement Workshop
Source: Implement Sci Commun. 2021 Apr 7;2:37. doi: 10.1186/s43058-021-00132-x (PMC8028199; doi:10.1186/s43058-021-00132-x)
Supplement: Supplementary file 4 — Additional file 4. Biweekly Facilitation Mini Interviews. [file 43058_2021_132_MOESM4_ESM.docx]

eScreening Implementation Pilot

Biweekly Facilitation Mini Interviews

Date:

1. What are some challenges you encountered regarding the implementation of eScreening at your site over the past 2 weeks?

_________________________________________________________________________________________________________________________________________________________________________________________________________________________________________________________________________________________________________________________________________________________________________________________________________________________________________

1. How have you addressed these challenges?

_________________________________________________________________________________________________________________________________________________________________________________________________________________________________________________________________________________________________________________________________________________________________________________________________________________________________________

1. Which components of the implementation strategy did you use during the past 2 weeks?

_________________________________________________________________________________________________________________________________________________________________________________________________________________________________________________________________________________________________________________________________________________________________________________________________________________________________________

1. How did you use them?

_________________________________________________________________________________________________________________________________________________________________________________________________________________________________________________________________________________________________________________________________________________________________________________________________________________________________________

1. How useful did you find these sections?

_________________________________________________________________________________________________________________________________________________________________________________________________________________________________________________________________________________________________________________________________________________________________________________________________________________________________________

Other Notes:

_________________________________________________________________________________________________________________________________________________________________________________________________________________________________________________________________________________________________________________________________________________________________________________________________________________________________________
