# Supplementary material for: Adaptation of a quality improvement approach to implement eScreening in VHA healthcare settings: innovative use of the Lean Six Sigma Rapid Process Improvement Workshop
Source: Implement Sci Commun. 2021 Apr 7;2:37. doi: 10.1186/s43058-021-00132-x (PMC8028199; doi:10.1186/s43058-021-00132-x)
Supplement: Supplementary file 5 — Additional file 5. Survey Results. [file 43058_2021_132_MOESM5_ESM.docx]

Table 1. eScreening implementation survey results.

| **Question** | **Site 1 (N=7)** | **Site 2 (N=3)** |
| --- | --- | --- |
| 1. Please rate the strength of eScreening in your opinion | | |
| Weak | 0 | 0 |
| Strong | 100 | 100 |
| 2. Senior leadership/clinical management in your organization: | | |
| 2.a Rewards clinical innovation and creativity to improve patient care | | |
| Disagree | 0 | 0 |
| Neither | 43 | 0 |
| Agree | 57 | 100 |
| *2b. Solicit opinions of clinical staff regarding decisions about patient care | | |
| Disagree | 14 | 0 |
| Neither | 43 | 0 |
| Agree | 43 | 100 |
| *2c. Seek ways to improve patient education and increase participation in treatment | | |
| Disagree | 14 | 0 |
| Neither | 14 | 0 |
| Agree | 71 | 100 |
| 3. The proposed practice changes with eScreening should be effective, based on current scientific knowledge |  |  |
| Disagree | 0 | 0 |
| Neither | 100 | 100 |
| Agree | 0 | 0 |
| 4. Staff members in your organization: | | |
| 4a. Have a sense of personal responsibility for improving patient care outcomes | | |
| Disagree | 0 | 0 |
| Neither | 0 | 0 |
| Agree | 100 | 100 |
| 4b. Cooperate to maintain and improve effectiveness of patient care | | |
| Disagree | 0 | 0 |
| Neither | 14 | 0 |
| Agree | 86 | 100 |
| 4c. Are willing to innovate and/or experiment to improve clinical procedures | | |
| Disagree | 0 | 0 |
| Neither | 14 | 0 |
| Agree | 86 | 100 |
| 4d. Are receptive to change in clinical procedures | | |
| Disagree | 0 | 0 |
| Neither | 43 | 0 |
| Agree | 57 | 100 |
| 5.Senior leadership/clinical management in your organization: | | |
| 5a. Provide effective management for continuous improvement of patient care | | |
| Disagree | 0 | 0 |
| Neither | 14 | 0 |
| Agree | 86 | 100 |
| *5b. Clearly define areas of responsibility and authority for clinical managers & staff | | |
| Disagree | 14 | 0 |
| Neither | 14 | 0 |
| Agree | 71 | 100 |
| *5c. Promote team building to solve clinical care problems | | |
| Disagree | 14 | 0 |
| Neither | 29 | 0 |
| Agree | 57 | 100 |
| *5d. Promote communication among clinical services and units | | |
| Disagree | 14 | 0 |
| Neither | 14 | 0 |
| Agree | 71 | 100 |
| 6.Senior leadership/clinical management in your organization: | | |
| 6a. Establish clear goals for patient care processes & outcomes | | |
| Disagree | 0 | 0 |
| Neither | 29 | 0 |
| Agree | 71 | 100 |
| *6b. Provide staff members with feedback/data on effects of clinical decisions | | |
| Disagree | 14 | 0 |
| Neither | 43 | 0 |
| Agree | 43 | 100 |
| 6c. Hold staff members accountable for achieving results | | |
| Disagree | 0 | 0 |
| Neither | 29 | 0 |
| Agree | 71 | 100 |
| 7.When there is agreement that change needs to happen: | | |
| *7a. We have the necessary support in terms of financial resources | | |
| Disagree | 14 | 67 |
| Neither | 43 | 0 |
| Agree | 43 | 33 |
| 7b. We have the necessary support in terms of training | | |
| Disagree | 0 | 0 |
| Neither | 29 | 33 |
| Agree | 71 | 67 |
| *7c. We have the necessary support in terms of facilities | | |
| Disagree | 0 | 100 |
| Neither | 29 | 0 |
| Agree | 71 | 0 |
| *7d. We have the necessary support in terms of staffing | | |
| Disagree | 14 | 33 |
| Neither | 14 | 67 |
| Agree | 71 | 0 |
| 8. The implementation plan for eScreening: | | |
| *8a. Identified specific roles & responsibilities | | |
| Disagree | 0 | 0 |
| Neither | 0 | 33 |
| Agree | 100 | 67 |
| *8b. Clearly described tasks and timelines | | |
| Disagree | 0 | 0 |
| Neither | 0 | 33 |
| Agree | 100 | 67 |
| 8c. Includes appropriate provider/patient education | | |
| Disagree | 0 | 0 |
| Neither | 0 | 0 |
| Agree | 100 | 100 |
| 8d. Acknowledges staff input & opinions | | |
| Disagree | 0 | 0 |
| Neither | 0 | 0 |
| Agree | 100 | 100 |
| 9. The proposed practice changes of eScreening are supported by clinical evidence with VA patients | | |
| Disagree | 0 | 0 |
| Neither | 0 | 0 |
| Agree | 100 | 100 |
| 10. The proposed practice changes: | | |
| 10a. Have been well-accepted by VA patients in a pilot study | | |
| Disagree | 0 | 0 |
| Neither | 0 | 0 |
| Agree | 100 | 100 |
| 10b. Are consistent with clinical practice that have been accepted by VA patients | | |
| Disagree | 0 | 0 |
| Neither | 0 | 0 |
| Agree | 100 | 100 |
| 10c. Take into consideration the needs and preferences of VA patients | | |
| Disagree | 0 | 0 |
| Neither | 0 | 0 |
| Agree | 100 | 100 |
| 10d. Appear to have more advantages than disadvantages for VA patients | | |
| Disagree | 0 | 0 |
| Neither | 0 | 0 |
| Agree | 100 | 100 |
| 11. I am familiar with the content and goals of eScreening | | |
| Disagree | 0 | 0 |
| Neither | 0 | 0 |
| Agree | 100 | 100 |
| 12. In my work, it is my responsibility to use eScreening | | |
| Disagree | 0 | 0 |
| Neither | 0 | 0 |
| Agree | 100 | 100 |
| 13. I have been trained in how to use eScreening | | |
| Disagree | 0 | 0 |
| Neither | 0 | 0 |
| Agree | 100 | 100 |
| 14. I have the skills to use eScreening | | |
| Disagree | 0 | 0 |
| Neither | 0 | 0 |
| Agree | 100 | 100 |
| *15. I am confident I can incorporate eScreening into my clinical care | | |
| Disagree | 0 | 0 |
| Neither | 14 | 33 |
| Agree | 86 | 67 |
| 16. I am confident that I can use eScreening even when others may not | | |
| Disagree | 0 | 0 |
| Neither | 14 | 0 |
| Agree | 86 | 100 |
| 17. I am confident I can use eScreening even when there is little time |  |  |
| Disagree | 0 | 0 |
| Neither | 14 | 0 |
| Agree | 86 | 100 |
| 18. For me, using eScreening is useful | | |
| Disagree | 0 | 0 |
| Neither | 0 | 0 |
| Agree | 100 | 100 |
| 19. For me, using eScreening is worthwhile | | |
| Disagree | 0 | 0 |
| Neither | 0 | 0 |
| Agree | 100 | 100 |
| 20. I intend to use eScreening in the next three months | | |
| Disagree | 0 | 0 |
| Neither | 0 | 0 |
| Agree | 100 | 100 |
| 21. I will definitely use eScreening in the next three months | | |
| Disagree | 0 | 0 |
| Neither | 0 | 0 |
| Agree | 100 | 100 |
| *22. eScreening is compatible with my work routine | | |
| Disagree | 0 | 0 |
| Neither | 14 | 33 |
| Agree | 86 | 67 |
| 23. eScreening has advantages compared to standard care | | |
| Disagree | 0 | 0 |
| Neither | 14 | 0 |
| Agree | 86 | 100 |
| 24. eScreening costs little time to deliver | | |
| Disagree | 0 | 0 |
| Neither | 0 | 0 |
| Agree | 100 | 100 |
| *25. Within my organization, all the necessary resources are available to properly use eScreening | | |
| Disagree | 0 | 0 |
| Neither | 20 | 67 |
| Agree | 80 | 33 |
| 26. The implementation team provided sufficient materials in using and maintaining eScreening | | |
| Disagree | 0 | 0 |
| Neither | 0 | 0 |
| Agree | 100 | 100 |
| 27. I believe that I can count on support from management when things get tough in using eScreening | | |
| Disagree | 0 | 0 |
| Neither | 0 | 0 |
| Agree | 100 | 100 |
| 28. I believe I can can count on support from peers who also use eScreening when things get tough | | |
| Disagree | 0 | 0 |
| Neither | 0 | 0 |
| Agree | 100 | 100 |
| 29. I have a clear plan how I will use eScreening | | |
| Disagree | 0 | 0 |
| Neither | 0 | 0 |
| Agree | 100 | 100 |
